# Quantitative and qualitative differences in anti-Envelope IgG-secreting cells distinguish subclinical and hospitalized dengue in a Cambodian pediatric cohort

**DOI:** 10.1371/journal.ppat.1014552

**Published:** 2026-10-01

**Authors:** Amandine Trouchet, Sokchea Lay, Matteo Broketa, Pablo Canales-Herrerias, Angga Perima, Bruno Iannascoli, Sotheary Sann, Borita Heng, Sowath Ly, Veasna Duong, Gaël A. Millot, Laura Walker, Patrick England, Giovanna Barba-Spaeth, Tineke Cantaert, Pierre Bruhns

**Affiliations:** 1 Institut Pasteur, Université Paris Cité, INSERM UMR1222, Antibodies in Therapy and Pathology, Paris, France; 2 Immunology Unit, Institut Pasteur du Cambodge, Pasteur Network, Phnom Penh, Cambodia; 3 Sorbonne University, Paris, France; 4 Epidemiology and Public Health Unit, Institut Pasteur du Cambodge, Pasteur Network, Phnom Penh, Cambodia; 5 Virology Unit, Institut Pasteur du Cambodge, Pasteur Network, Phnom Penh, Cambodia; 6 Institut Pasteur, Université Paris Cité, Bioinformatics and Biostatistics Hub, Paris, France; 7 Moderna, Inc., Cambridge, Massachusetts, United States of America; 8 Institut Pasteur, Université Paris Cité, CNRS UMR3528, Platform of Molecular Biophysics, Paris, France; 9 Institut Pasteur, Université Paris Cité, CNRS UMR 3569, Structural Virology, Paris, France; 10 Institut Pasteur, Université Paris Cité, CNRS UMR 2000, Ecology and Emergence of Arthropod-borne Pathogens, Paris, France; SUNY Upstate Medical University: State University of New York Upstate Medical University, UNITED STATES OF AMERICA

## Abstract

Dengue virus (DENV) infection can result in outcomes ranging from subclinical infection to life-threatening illness, yet the antibody features mediating protection in humans remain poorly defined. Dengue virus envelope protein (E) forms homodimers on the virion surface and elicits both neutralizing and non-neutralizing antibodies from IgG-secreting cells (IgG-SC). How the developing antibody response at the monoclonal level contributes to protection is unknown. We profiled single IgG-SC responses in a Cambodian pediatric cohort by measuring affinity and serotype cross-reactivity of secreted antibodies from patients with subclinical or hospitalized dengue during acute DENV1 or DENV2 infection. Using droplet-based microfluidic imaging, we analyzed over 3,300 single IgG-SC. Hospitalized patients exhibited significantly lower numbers of IgG-SC, despite comparable IgG secretion rates and similar frequencies of DENV E-specific antibody-secreting cells relative to subclinical cases. In hospitalized patients, the affinity of IgG-SC for the DENV1 E-dimer was 3-fold lower compared to subclinical cases. Notably, antibodies recognizing quaternary epitopes—binding E-dimers but not monomers—were detected exclusively in subclinical cases, whereas hospitalized patients predominantly produced monomer-reactive antibodies. These findings suggest that dengue severity may be linked to the inefficient generation of sufficient numbers of high-affinity, broadly reactive antigen-specific IgG-SC during post-primary infection.

## Author summary

Most dengue virus infections cause mild illness or no symptoms, but some individuals progress to severe disease requiring hospitalization. Understanding why these outcomes differ is essential for improving vaccines and treatments. Antibodies play a central role in controlling dengue infection, but not all antibodies are equally protective. We examined antibody-producing cells generated during acute dengue infection in Cambodian children with subclinical infection or hospitalized dengue. Using a high-throughput single-cell microfluidic platform, we analyzed more than 3,300 individual IgG-secreting cells and measured the strength and specificity of the antibodies they produced. Hospitalized children generated similar proportions of dengue-specific antibody-producing cells, but they had fewer of these cells and their antibodies bound the viral envelope protein with lower affinity than those from children with subclinical infection. Importantly, only children with subclinical infection produced antibodies that recognized complex three-dimensional (quaternary) structures on the virus surface, which are thought to be among the most effective at neutralizing dengue virus. Our findings highlight the importance of monitoring antibody responses at the single-cell level and considering antigen structure in dengue vaccine design to promote protective immunity while reducing the risk of disease-associated responses.

## Introduction

Dengue is the most widespread arboviral disease, and about half of the global population lives in areas at risk of dengue infection [1,2]. Global incidence has almost doubled in the last 3 decades and is expected to continue growing in Latin America, Asia and sub-Saharan Africa. After the bite with a dengue virus (DENV)-infected Aedes mosquito, the disease outcome is variable. Most individuals undergo either asymptomatic infection with no symptoms, inapparent infection, which is defined as a very mild disease that does not disturb the daily activities of the patient, or uncomplicated self-resolving febrile illness [3–5]. Approximately 10% of all dengue infections can lead to hospitalization [6,7]. Severe dengue is characterized by plasma leakage, which can be accompanied by hemorrhagic symptoms.

The disease can be caused by one of the 4 closely related serotypes, DENV1–4. As all 4 serotypes co-circulate in endemic areas, individuals can be infected with multiple serotypes throughout their lives. Infection with one serotype induces short-term serotype cross-reactive protection from disease and can confer life-long protection against symptomatic homotypic re-infection, albeit homotypic symptomatic repeat infections have been reported [8–11]. In contrast, secondary heterotypic infection is a risk factor for severe dengue [6,12,13]. It is hypothesized that immune complexes of DENV and anti-dengue antibodies mediate antibody dependent enhancement (ADE), where myeloid cells are infected following binding of the immune complexes to Fcγ receptors (FcγR) [14,15]. This could result in increased infected cell mass and in the suppression of anti-viral immunity and altered inflammatory responses [16–18]. ADE is thought to be mediated by non-neutralizing anti-dengue antibodies. ADE occurs when antibody concentrations are waning and when either cross-reactive low affinity antibodies, and/or antibodies targeting specific epitopes are present [19–21], and depends on the time, antigenic distance and order of previous DENV infections [22,23]. In addition, variations of the glycan structure bound to IgG Fc that enhance FcγR binding lead to enhanced ADE and are associated with more severe disease [14,24,25].

The antibody response to DENV is mainly directed towards the dimeric envelope (E) protein. The monomeric soluble form of the E protein (sE) is used for antibody detection in the patient’s serum for diagnosis. sE exposes the fusion loop epitope (FLE). The FL epitope is immunodominant, as the FL is conserved between DENV serotypes and other flaviruses [26,27]. Anti-FL antibodies are broadly cross-reactive but poorly neutralizing, and display a strong infection enhancing potential, i.e., ADE [21,26–30]. In contrast, antibodies binding to quaternary epitopes on E proteins have low ADE potential and are broadly cross-neutralizing [27,30–32]. We previously engineered sE-dimers locked by inter-subunit disulfide bonds to recapitulate quaternary epitopes found on the surface of the dengue virion, while masking the FL epitope [33].

In an acute post-primary infection, activated memory B cells rapidly proliferate and differentiate into circulating plasmablasts and plasma cells, which here we collectively term IgG-secreting cells (IgG-SC). Circulating plasmablast numbers peak at day 6–7 post onset of symptoms, coinciding with the period where patients are at risk of developing severe symptoms. Studies by us and others have shown that a highly expanded plasmablast compartment correlated with severe secondary dengue [34–36]. The antibodies generated by these cells will influence the disease’s progression, potentially offering protection or, conversely, contributing to ADE. Although extensive research has characterized the specificity, affinity, and cross-reactivity of the polyclonal serum response following natural dengue infection in hospitalized patients or following vaccination, far fewer studies have examined the developing antibody response at the monoclonal level and how it relates to protective immunity [37–40].

We have previously developed a droplet-based microfluidic technology (DropMap) which allows for quantitative and high-throughput analysis of individual IgG-SC [41]. DropMap combines the immobilization within an observation chamber of ~100,000 picoliter-size droplets containing non-sorted single cells with an ultrasensitive fluorescent bioassay [42]. DropMap was reported for clinical studies to measure single-cell cytokine secretion from sepsis patients [43], and affinity and secretion rate of IgGs secreted by autoreactive IgG-SC from blood, spleen and bone marrow of immune thrombocytopenia patients [44], and by IgG-SC from SARS-CoV-2 mRNA vaccinated donors [45]. Here, we describe the adaptation of the assay to study affinity and cross-reactivity to multiple dengue E proteins in a pediatric cohort of subclinical cases and hospitalized patients infected with either DENV1 or DENV2.

## Results

### Characterization of subclinical and hospitalized dengue patients

To understand the contribution of the anti-E antibody response to protection or disease during post-primary DENV infection, we identified subclinical acute dengue infected cases via a household-based approach [46]. In the current study, we included samples from 6 subclinical (patients S1 to S6) and 9 hospitalized (patients H1 to H9) dengue pediatric patients undergoing post-primary DENV1 or DENV2 infection collected in Kampong Cham Province in 2018 (Tables 1 and 2 and Fig 1A). Subclinical cases were confirmed by RT-qPCR positive for dengue, but did not meet the WHO 2009 classification criteria for dengue. All individuals showed at least one symptom. The mild disease presentation allowed us to select patients with similar time of disease onset as the hospitalized cases (inclusion, i.e., D0 corresponds to 2–3 days after onset of symptoms), ensuring that analyses were performed at similar timepoints in the disease dynamics in both groups.

**Table 1:** Demographic and clinical characteristics of the cohort.

Cohort	Hospitalized	Subclinical	All dengue cases	Healthy controls
	n = 9	n = 6	n = 15	n = 3
**Age**				
Mean (SD)	12 (4)	11 (4)	11 (4)	10 (5)
**Gender**				
Female	3 (33%)	1 (17%)	4 (27%)	1 (33%)
Male	6 (67%)	5 (83%)	11 (73%)	2 (67%)
**Infecting serotype**				
DENV1	3 (33%)	3 (50%)	6 (40%)	N/A
DENV2	6 (67%)	3 (50%)	9 (60%)	N/A
**Immune status**				
Post-primary	8 (89%)	6 (100%)	24 (96%)	N/A
Undetermined	1 (11%)	0 (0%)	1 (4%)	N/A
**Day of fever at inclusion**				
Mean (SD)	3 (2)	3 (3)	2 (2)	N/A
**WHO 2009 classification**				
Subclinical	0 (0%)	6 (100%)	6 (40%)	N/A
Uncomplicated dengue	4 (44%)	0 (0%)	4 (27%)	N/A
Dengue with warning signs	5 56%)	0 (0%)	5 (33%)	N/A

N/A: not applicable.

**Table 2:** Demographics of the patients.

Patient#	Sex	Age	Severity	Infecting serotype	# of IgG-SC analyzed	# of anti-E protein IgG-SC identified
**S1**	M	14	subclinical	**DENV1**	377	124
**S2**	M	2	subclinical	**DENV1**	302	138
**S3**	M	7	subclinical	**DENV1**	52	41
**S4**	M	12	subclinical	DENV2	816	323
**S5**	M	11	subclinical	DENV2	376	220
**S6**	M	15	subclinical	DENV2	574	327
**H1**	F	9	DWWS	**DENV1**	57	14
**H2**	M	14	D	**DENV1**	110	23
**H3**	F	5	DWWS	**DENV1**	29	9
**H4**	F	6	D	DENV2	111	88
**H5**	M	14	D	DENV2	26	8
**H6**	M	14	DWWS	DENV2	40	17
**H7**	M	13	DWWS	DENV2	69	42
**H8**	M	12	D	DENV2	5	5
**H9**	M	14	DWWS	DENV2	36	14

D: uncomplicated dengue, DWWS: dengue with warning signs.

**Fig 1:**
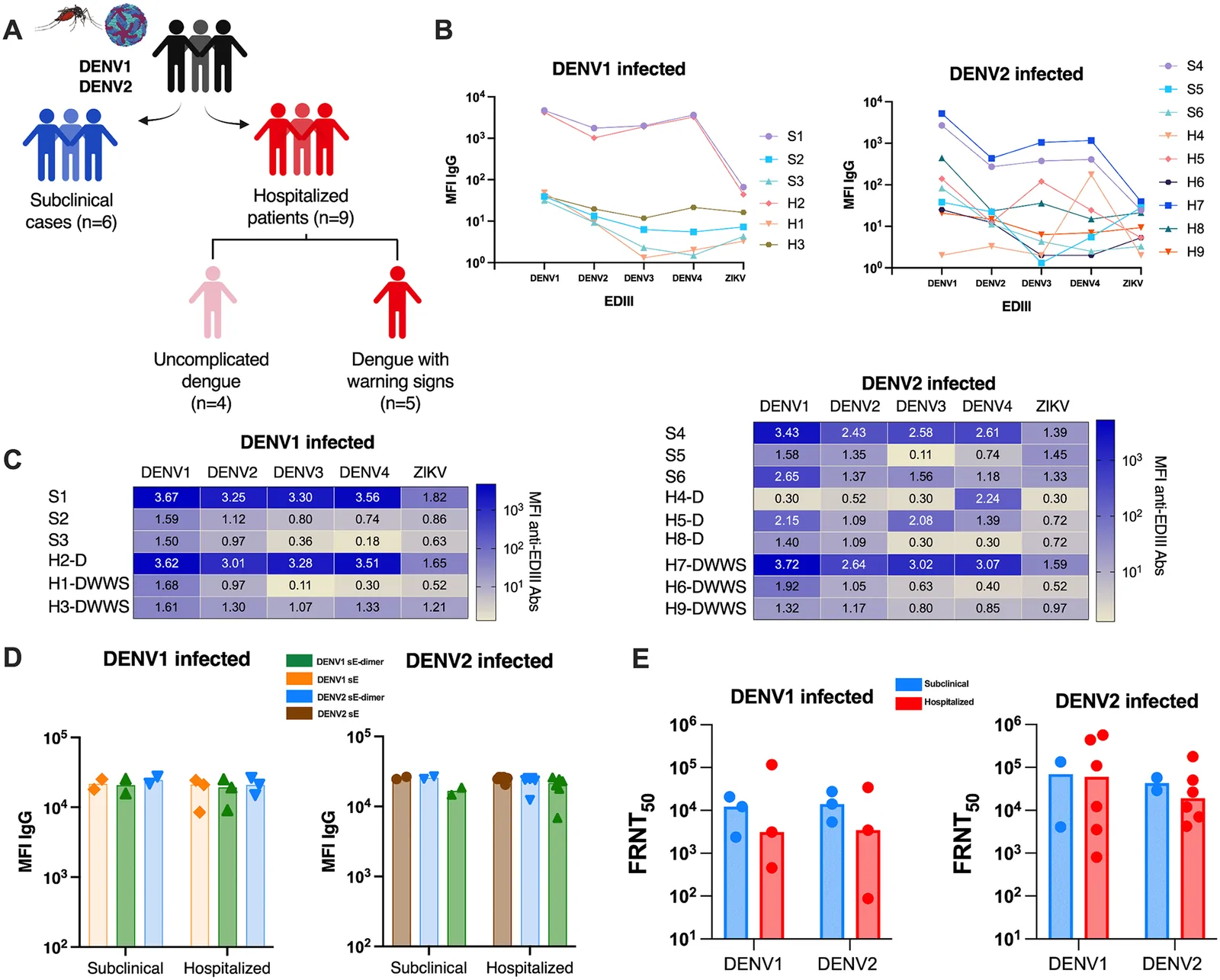
Characterization of the anti-E protein antibody response in plasma of subclinical and hospitalized post-primary DENV infected patients. **A.** Inclusion scheme of individuals with subclinical (blue, n = 6) and hospitalized (red, n = 9) post-primary DENV1 or DENV2 infections. Created in Biorender. **B.** Mean Fluorescence Intensity (MFI) of IgG binding to EDIII derived from DENV1-4 and ZIKV of plasma samples of subclinical (S: n = 6; numbered S1 to S6) and hospitalized (H; n = 9; numbered H1 to H9) individuals infected with DENV1 (left) or DENV2 (right) at day of inclusion **C.** Heatmap representation of MFI of IgG binding to EDIII derived from DENV1-4 and ZIKV. Each row represents one individual. Hospitalized patients are subcategorized as individuals with uncomplicated dengue (D), n = 4 or dengue with warning signs (DWWS), n = 5. **D.** Mean Fluorescence Intensity of IgG binding to DENV1 sE-dimer (green) or sE (orange), or DENV2 sE-dimer (blue) or sE (brown) in day 5 plasma samples of subclinical (n = 4) and hospitalized (n = 9) individuals infected with DENV1 (left) or DENV2 (right). IgG binding was measured by using a multiplex microsphere immunoassay (MIA) and are represented as median fluorescence intensity (MFI). **E.** Neutralizing antibody titers against DENV1 and DENV2 were compared between subclinical cases (blue; n = 5) and hospitalized patients (red; n = 9) on D5 plasma and the data shown as focus reduction neutralization test 50 (FRNT50). Each point represents an individual; for 2 subclinical cases, not sufficient plasma was retrieved to complete all the assays. Median and 95% confidence interval are shown.

Since these samples originated from a cross-sectional study, we do not have information on the infection history of these individuals. Hence, to gain insight into the previous infection serotype, we analyzed the anti-EDIII antibody response to the 4 DENV serotypes and the closely related Zika virus (ZIKV) of the individuals at inclusion [23]. Binding to anti-EDIII from DENV1 tended to be higher than to EDIII of the other serotypes in the majority of individuals, irrespective of the current infecting serotype (patients S2, S3, H1, S4, S5, S6, H6, H and H8), except for one individual with a strong EDIII DENV-4 binding preference (patient H4), and some individuals where the preferential binding serotype was less clear (patients S1, H2, H3, H5 and H9) (Fig 1B and 1C). Together with the age profile of the subjects (mean age of 12 years old in 2018), and the high DENV1 circulation from 2010 to 2016 in Cambodia [47], these data suggest that the patients included had prior DENV1 infection, apart from 1 hospitalized patient infected with DENV2 who may have been more likely to have had a prior DENV4 infection.

Next, we characterized the patients’ anti-DENV E protein antibody response by multiplex immunoassay using sE and sE-dimer proteins of Cambodian isolates of DENV1 and DENV2 and by seroneutralization assays. Antibodies from patient plasma bound to both recombinant sE and sE-dimers of both serotypes, and no difference in titers or neutralization potential was observed in subclinical or hospitalized patients, albeit the patient sample size was small (Fig 1D and 1E).

Thus, we identified 6 subclinical and 9 hospitalized dengue pediatric patients with pre-existing dengue immunity for the analysis of the IgG-SC compartment during post-primary infection.

### Single-cell bioassay allows phenotypic characterization of dengue-specific IgG-SC

For this study, we adapted a single-cell bioassay in microfluidic droplets termed “DropMap” that we have described previously [41,44,45]. DropMap allows for a direct sorting-free assessment of the antibody-based cellular immune response, while characterizing the secretion rate, specificity, and affinity for sE and sE-dimer from DENV of human IgG secreted by IgG-SC, allowing for analysis of 10,000–20,000 cells/hour.

Here, human peripheral blood mononuclear cells (PBMCs) of DENV-infected children obtained 5 days after RT-PCR confirmed infection, corresponding to day 7–8 after onset of symptoms in both hospitalized and subclinical cases, were investigated. PBMC were co-encapsulated in droplets along with paramagnetic nanoparticles and fluorescent bioassay reagents that were immobilized within an observation chamber and imaged over 1 hour by time-lapse fluorescence imaging (Figs 2A and S1A). Including paramagnetic beads in the droplets allows for the use of a magnetic field to align them and make a clearly distinguishable pattern (the “beadline”) by microscopy. Because the beads are coated with an IgG capture reagent, the beadline serves herein as a physical surface for a time-resolved double-fluorescent sandwich immunoassay, revealing both IgG secretion inside the droplet and specificity of the produced IgG for three dengue proteins (Fig 2A). The DENV1 bioassay consisted of DENV1 sE-dimer, DENV2 sE-dimer and DENV1 sE monomer, whereas the DENV2 bioassay consisted of DENV1 sE-dimer, DENV2 sE-dimer and DENV2 sE monomer. IgG secretion rates from each IgG-SC were estimated using calibration curves generated with human monoclonal IgGs (Table 3) at an average of 10–20 IgG molecules per second (with variation from 5-250 molecules/s) [45].

**Table 3:** Characteristics of the mAbs used for the generation of the calibration curves.

Name	Reference	Targets (serotypes bound)	K_D_ (M)			
			DENV1 sE	DENV1 sE-dimer	DENV2 sE	DENV2 sE-dimer
ADI-24191	ADImab	EDIII (DENV1)	1.30 E-10	1.10 E-10	N.B.	N.B.
ADI-24229	ADImab	EDIII (DENV1)	1.50 E-10	2.70 E-10	N.B.	N.B.
ADI-24197	ADImab	EDIII (DENV1)	3.00 E-09	2.10 E-09	N.B.	N.B.
6FLA	[48]	EDIII (DENV2)	N.B.	N.B.	7.10 E-11	1.60 E-10
6FLC	[48]	EDIII (DENV2)	N.B.	N.B.	6.90 E-11	3.60 E-10
1A1D-2	[49]	EDIII (DENV1–3)	2.10 E-10	1.60 E-10	1.80 E-10	2.80 E-10
5AAM	[50]	EDIII (DENV1–4)	2.60 E-10	4.10 E-10	2.20 E-10	5.10 E-10
ADI-24269	ADImab	E (DENV1–4)	3.90 E-10	3.40 E-10	3.10 E-10	2.40 E-10
ADI-24194	ADImab	E (DENV1–4)	1.20 E-09	1.10 E-09	7.90 E-10	1.10 E-09
ADI-24292	ADImab	E (DENV1–4)	1.20 E-09	1.10 E-09	4.70 E-08	1.60 E-09
Negative control (anti-SARS-Cov-2 mAb clone 210)	in house	irrelevant	2.90 E-07	6.80 E-08	1.60 E-07	3.60 E-07

List of mAbs used for the calibration curves represented in S2 Fig. N.B. non-binding.

**Fig 2:**
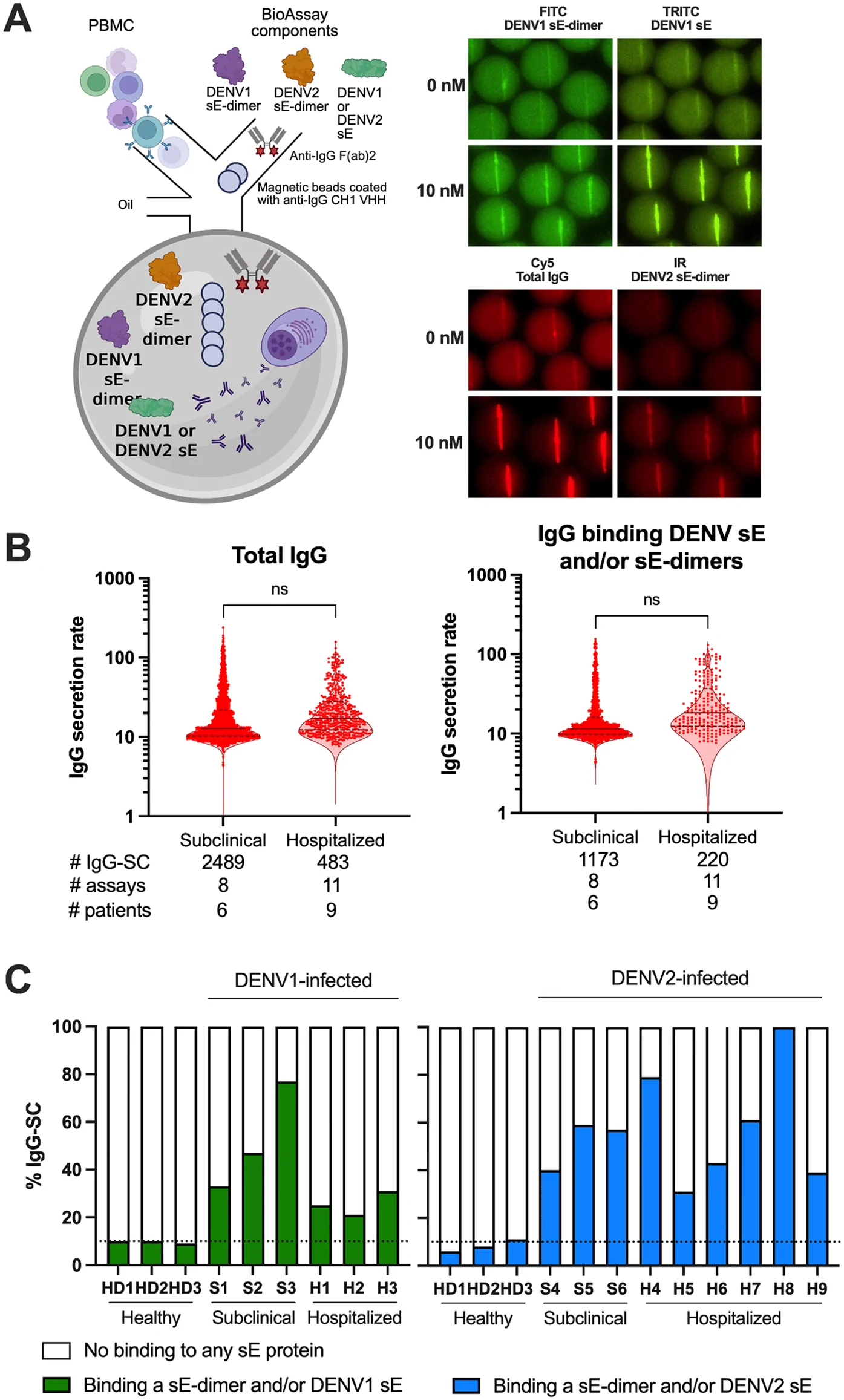
Significantly lower IgG-SC numbers in hospitalized patients compared to subclinical cases. **A.** (Left) Schematic representation of the DropMap bioassay. Created in Biorender. (Right) Representative images of the DropMap bioassay including relocation onto the beadline in droplets of DENV1 sE-dimer (FITC), DENV1 sE (TRITC) and DENV2 sE-dimer (IR) in the absence (0 nM; negative control) or presence of anti-DENV1/DENV2 E protein mAb ADI24-294 (10nM; positive control). **B.** IgG secreting rate from (left) total IgG-SC or from (right) IgG-SC binding to sE and/or sE-dimer of DENV1 or DENV2 in PBMCs from hospitalized dengue patients and subclinical cases at day 5 post RT-qPCR confirmed DENV infection, corresponding to day 7-8 after onset of symptoms. Horizontal bars represent median and IQR. Each dot represents one IgG-SC. *p* values were calculated using two-by-two contrast comparisons after linear modelling. ns, not significant**. C.** Proportion of IgG-SC not binding any DENV E protein (non-specific IgG-SC; white boxes), or binding at least one DENV1 E protein (to sE and/or sE-dimer; green boxes), or binding at least one DENV2 E protein (to sE and/or sE-dimer; blue boxes) in age-matched Cambodian healthy donors (HD) or in individuals infected with either (left) DENV1 or (right) DENV2. Patients with subclinical dengue (S) or hospitalized dengue (H) were numbered and stratified by infecting DENV serotype.

Taken together, we have setup a bioassay that allows for the evaluation of antibody affinity and cross-serotype binding properties of the newly developing anti-E antibody repertoire during post-primary infection.

### Evaluation of secretion rate and antigen specificity of single IgG-SC

No difference in total IgG secretion rate or in sE-specific IgG secretion rate was observed between DENV seronegative Cambodian children, which acted as negative healthy donors (HD), and DENV-infected cases, whether subclinical or hospitalized (S1B Fig). Samples from hospitalized cases showed a modest (~1.5-fold) higher secretion rate compared to samples from subclinical cases (Total IgG: 17.9 (12.29-27.28) versus 12.75 (10.28-21.87) molecules/s (Fig 2B), but this difference was mainly due to a patient effect (evidenced by two-by-two contrast comparisons after linear modelling of single patient data; S2 Fig). Flow cytometry analyses of the same samples also showed 3-fold lower frequencies of CD19^+^IgD^-^IgM^-^IgG^+^ B cells among CD19^+^CD3^-^ cells in hospitalized compared to subclinical patients, and similar frequencies of CD19^+^IgD^-^CD27^+^CD38^++^ plasmablasts (S1C and S1D Fig). As expected, we could only detect few sE-specific IgG-SC in the HD group.

DropMap also allows the affinity of a secreted IgG within each droplet to be estimated for the fluorescently labeled dengue proteins that make up the assay using calibration curves generated with monoclonal anti-DENV1 sE-dimer, anti-DENV2 sE-dimer and anti-sE IgG with known affinity (K_D_), as we have previously reported for different antigens [44,45,51]. However, because secreted IgG may bind one, two or even all three of these dengue antigens present in each droplet, competition for the target antigen occurs. We thus generated reference curves for each of these scenarios (S3 Fig), applying the corresponding reference curve once the mono- or cross-specificity of the IgG-SC had been established. The resulting reference curves generated using 11 different anti-dengue IgG mAbs of various affinities (Table 3) allow for estimation of affinities over 2 logs of affinities, i.e., 2x10^-10^ ≤ K_D_ ≤ 3x10^-8^ M (S3 Fig). Importantly, Table 3 shows that several cross-reactive calibration mAbs displayed comparable affinities across the E-derived antigens tested, limiting the risk that the calibration curves were driven only by strongly unbalanced competition toward a single antigen. Therefore herein, IgG interacting with dengue antigens sE-dimer or sE at a calculated K_D_ below 3x10^-8^ M (30 nM) will be considered binding, i.e., anti-dengue IgG antibodies, and the cell secreting such IgGs will be termed dengue-specific IgG-SC. Affinities calculated from DropMap data better than 2x10^-10^ M were mathematically extrapolated and therefore approximated. As expected, the percentages of IgG-SC binding to at least one DENV sE protein was lower (3–8-fold) in HD compared to DENV-infected individuals (Fig 2C). The percentage of IgG-SC binding to at least one DENV sE protein among all IgG-SC varied from 33 to 77% (mean 52%) in subclinical cases and from 20 to 100% (mean 47%) in hospitalized patients (Fig 2C); thus, the proportion of anti-sE protein IgG-SC within all IgG-SC analyzed was highly variable among patients, but overall similar in hospitalized patients compared to subclinical cases.

In conclusion, hospitalized patients displayed significantly lower numbers of IgG-SC, despite secreting IgG at similar rates and exhibiting comparable frequencies of DENV E-specificity compared to subclinical cases.

### Evaluation of affinity and serotype cross-reactivity of single IgG-SC

Next, we aimed at understanding the affinity and cross-reactivities of antibodies recognizing one or both sE-dimers in subclinical cases (n = 6) and hospitalized cases (n = 9). Affinities for sE-dimers were detected from 30nM (the limit of detection) to ~1 pM, ranging over more than 3 logs (Fig 3). In subclinical and hospitalized patients infected with DENV1, IgG-SC recognizing both DENV1 sE-dimers and DENV2 sE-dimer had significantly higher affinity for DENV1 sE-dimer (>10-fold) and DENV2 sE dimer (>2-fold) compared to IgG-SC only recognizing DENV1 sE-dimer or DENV2 sE-dimer, respectively, even when considering potential patients’ effects (Fig 3A and 3B). Interindividual variations were sometimes significant among subgroups, but for all but one individual (patient S5) the higher affinities for DENV1-sE dimer observed for IgG-SC recognizing both dimers were also significantly higher than affinities of IgG-SC recognizing a single dimer when analyzing individual subclinical patient data; hospitalized patient IgG-SC data was scarce and mainly supported this trend in affinities (S4A and S4B Fig). Affinities of IgG-SC recognizing both dimers were also significantly better than affinities of IgG-SC recognizing a single dimer in subclinical and hospitalized patients infected with DENV2 (Fig 3C and 3D) with, again, some significant differences among individual patient responses but a conserved trend (S4C and S4D Fig). Finally, the mean affinity of IgG secreted by IgG-SC recognizing both sE-dimers was significantly higher for DENV1 sE-dimer than for DENV2 sE-dimer in DENV1- and DENV2-infected patients, even when considering potential patient effects (Fig 3A and 3C), suggestive that the suspected previous infecting serotype was indeed DENV-1 for most patients analyzed, as supported by the increased binding to EDIII of DENV1 in the acute phase plasma (Fig 1B).

**Fig 3:**
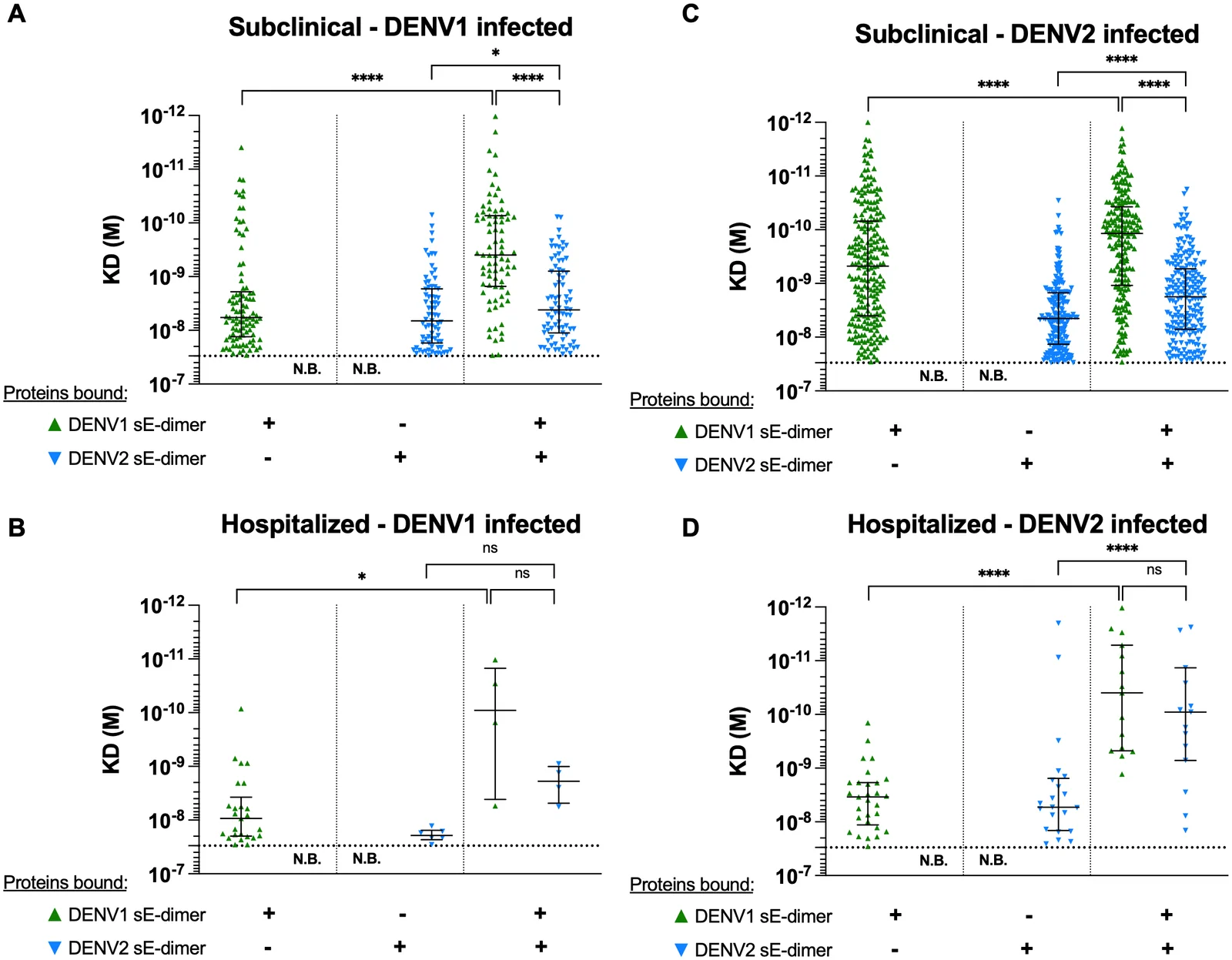
Dengue infected patients display IgG-SC of higher affinity for DENV1 sE-dimers than DENV2 sE dimers irrespective of the infecting serotype. **(A-B)** DropMap-measured affinity (K_D_) of IgG-SC from **(A)** subclinical or **(B)** hospitalized patients infected with DENV1. **(C-D)** DropMap-measured affinity (K_D_) of IgG-SC from **(C)** subclinical or **(D)** hospitalized patients infected with DENV2. **(A-D)** Data are divided by categories of IgG-SC that bind one sE-dimer or both sE-dimers, as indicated in the legend below the graphs. Horizontal bars indicate median and interquartile range. Each dot represents the K_D_ value of one IgG-SC recognizing at least one sE-dimer. *p* values were calculated using two-by-two contrast comparisons after linear modelling. ns, not significant; *, p ≤ 0.05; ****, p ≤ 0.0001. The dotted line indicates the 30nM affinity limit that discriminates E protein binders from non-binders. N.B.: non-binding.

As our assay could accommodate a maximum of 3 antigens, we decided to perform a DropMap experiment twice per individual, once including as antigens sE and sE-dimer of the infecting serotype, and the sE-dimer of the non-infecting serotype (termed “infection assay”) and once including as antigens sE and sE-dimer of the non-infecting serotype and sE-dimer of the infecting serotype (termed “cross assay”). This setup allowed for the determination of serotype cross-reactive IgG-SC and served as biological replicates in the determination of IgG-SC specific for the sE-dimers of both DENV1 and DENV2 in case sufficient PBMC were available. The cross-assay validated the approach and reproducibility of the assays (S5 Fig).

Taken together, subclinical cases displayed IgG-SC of higher affinity for DENV1 sE-dimers than DENV2 sE dimers irrespective of the infecting serotype.

### Evaluation of epitope specificity of single IgG-SC

Next, we compared numbers of dengue-specific IgG-SC and the affinity of their secreted antibodies that bind to at least one DENV sE or sE-dimer protein in subclinical (n = 6) and hospitalized (n = 9) dengue patients and in DENV seronegative Cambodian children (HD; n = 3). As expected, the few IgG-SC of the HD binding to the sE-derived antigens showed significantly lower binding affinity (K_D_ ~ 10^-8^ or worse) compared to DENV-infected patients (S6 Fig). In patients infected with DENV1, dengue-specific IgG-SC from subclinical patients demonstrated at least 3-fold higher affinity for DENV1 sE-dimer compared to hospitalized patients (median: 2x10^-9^ versus 8x10^-9^ M, p = 0.0029, respectively), but not towards the cross-reactive dimer, i.e., DENV2 sE-dimer (Fig 4A). This difference was also observed on a patient level (S7A Fig) with, nevertheless, inter-patient variations explaining part of the observed differences when integrating the protein bound, clinical status and random effect of patients (S7B Fig). Higher affinity for DENV1 sE-dimer was also observed for IgG-SC from DENV2-infected subclinical compared to hospitalized patients (Fig 4A) but was mainly explained by inter-patient variations (S7A and S7B Fig).

**Fig 4:**
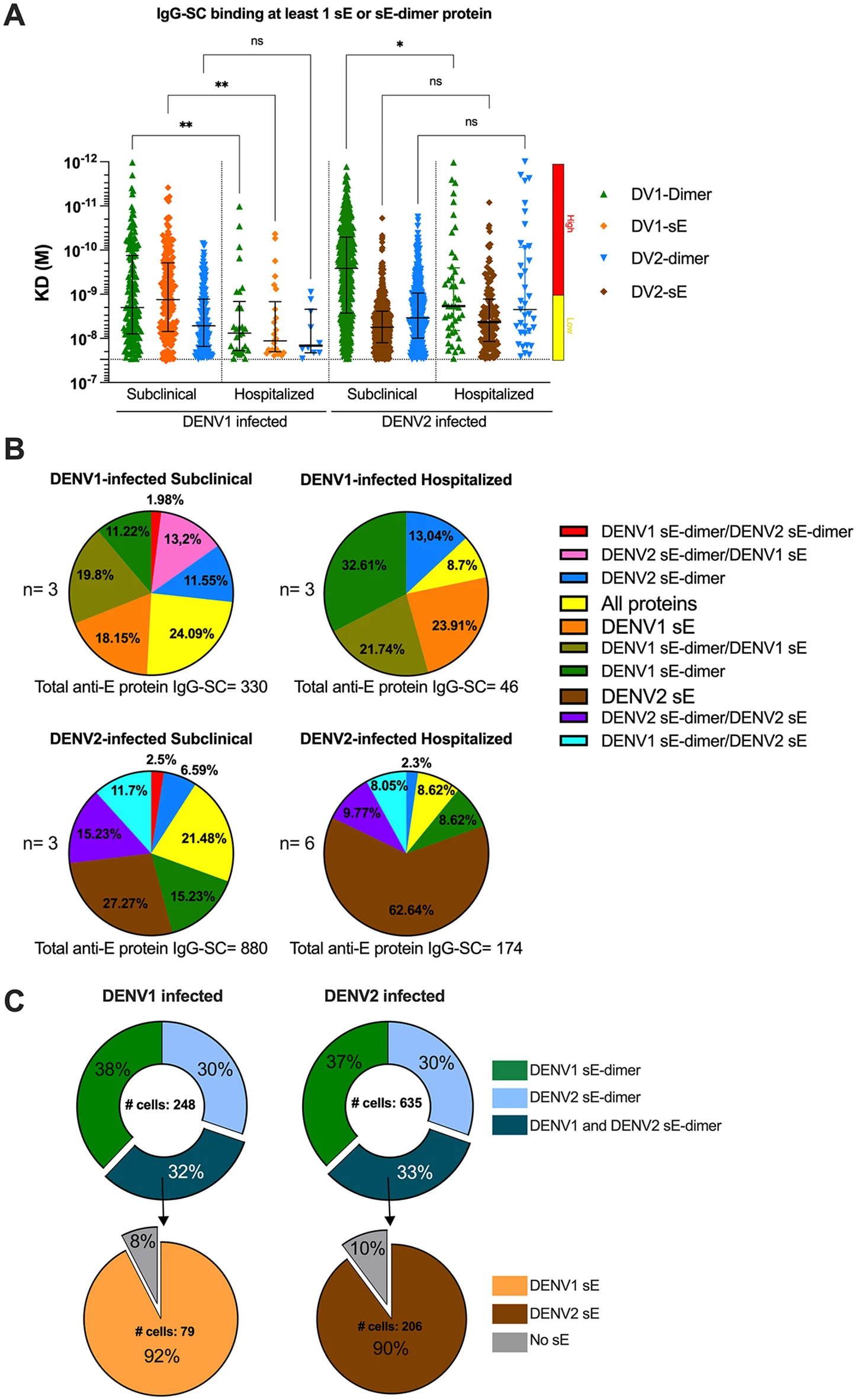
Subclinical cases display IgG-SC of higher affinity for DENV1 sE and sE-dimer, and higher frequencies of serotype cross-reactive IgG-SC compared to hospitalized patients. **A.** DropMap-measured affinity (K_D_) of sE-protein-specific IgG-SC binding in subclinical or hospitalized patients infected with (left) DENV1 or (right) DENV2. Horizontal bars indicate median and interquartile range. Each dot represents one sE-protein-specific IgG-SC. *p* values were calculated using Kruskall-Wallis followed by Dunn’s multiple comparison test. ns, not significant; *, p ≤ 0.05; **, p ≤ 0.01. The dotted line indicates the 30nM affinity limit that discriminates E protein binders from non-binders. **B.** Proportions of IgG-SC binding to the different sE and/or sE-dimer proteins in both patient groups. Number of patients (n), total number of anti-E protein IgG-SC analyzed and percentages of anti-E protein IgG-SC falling in each category are indicated. **C.** (Top) Frequencies of IgG-SC binding to sE-dimer of only one serotype or both serotypes in subclinical infected cases. (Bottom) Frequencies of IgG-SC with (orange or brown) detectable or (grey) undetectable binding to sE monomers by DropMap from the IgG-SC binding both serotypes (from the dark blue slice) in subclinical infected cases. Frequencies are shown.

Similarly, the affinity of antibodies towards DENV1 sE monomer was 10-fold higher in subclinical patients compared to hospitalized patients infected with DENV1 (1x10^-9^ vs 1x10^-8^ M, p = 0.0005, respectively), whereas no difference in affinity was observed towards DENV2 sE monomer between subclinical and hospitalized patients infected with DENV2 (Fig 4A). Inter-patient variations explained part of the observed differences (S7A and S7B Fig).

To identify dengue-specific IgG-SC producing cross-reactive antibodies to DENV1 and DENV2 sE proteins and those producing antibodies specifically targeting quaternary epitopes on the sE-dimer surface, we evaluated the proportion of antibodies binding to multiple antigens. In DENV2-infected hospitalized patients, more dengue-specific IgG-SC secreted antibodies binding only to DENV2 sE than in subclinical patients (27% vs 62%, DENV2 sE: brown) (Fig 4B). This difference in IgG-SC binding preference to DENV2-sE in DENV2-infected patients was also evidenced on a patient level (S8 Fig). In contrast, more IgG-SC bound to all three proteins (both sE-dimers and sE, yellow) in subclinical patients compared to hospitalized patients (DENV1 cohort: 24% vs 9%; DENV2 cohort 21% vs 9%) with, however, notable interpatient variations. Importantly, dengue-specific IgG-SC secreting cross-reactive antibodies binding to quaternary epitopes - thus binding to sE-dimer but not sE monomers - could only be detected in subclinical patients (Figs 4B and S8, referred to as “DENV1 sE-dimer/DENV2 sE-dimer”, red). In both the DENV1 and DENV2 infected patients, one third of the dengue-specific IgG-SC recognized the DENV1 sE-dimer specifically, one third the DENV2 sE-dimer specifically, and one third recognized both dimers. From these latter IgG-SC recognizing both sE-dimers, 10% did not recognize sE monomers, indicating these rare cells (2.5-3% of sE-dimer binding cells) are the dengue-specific IgG-SC binding quaternary epitopes (Fig 4C).

Hence, IgG-SC from subclinical cases showed a higher affinity for DENV1 sE-dimer compared to hospitalized patients, with strong inter-patient variations. In addition, antibodies targeting quaternary antibodies could exclusively be found in subclinical cases, while hospitalized patients show a higher frequency of IgG-SC specific for dengue sE antigens.

## Discussion

Whereas antibodies generally are thought to play a protective role after viral infection, it has become clear that antibodies targeting DENV E protein can play a protective or pathogenic role during a subsequent heterotypic DENV infection. DENV-specific antibodies can either neutralize the virus or induce immunopathogenic mechanisms depending on the titer, affinity, avidity and targeted epitopes on the E protein [19,20,25,28,32]. During acute post-primary infection, both pre-existing anti-DENV IgG and newly formed IgG produced by IgG-SC co-circulate. These IgG-SC are either directly derived from memory B cells, retaining binding specific for the previous infecting serotype or are the result of recall germinal center reactions inducing further maturation and selection [52–54]. Studies by us and others have shown that a highly expanded plasmablast compartment correlates with severe dengue [34–36,55–57]. Interestingly, plasmablasts are also highly expanded in patients with other systemic infections causing hemorrhagic fevers, such as Ebola [58]. In this work, we compared IgG-secreting plasmablast populations from pediatric cohorts based on Cambodian children hospitalized for dengue with their siblings or close neighbors undergoing a subclinical infection (household cohort). We found that hospitalized children had similar frequencies of IgG-SC binding to sE and/or sE dimer within the total IgG-SC population compared to children with subclinical symptoms. However, sE-protein-specific IgG-SC from subclinical cases were of higher affinity for sE and sE-dimer with some IgG-SC targeting quaternary epitopes, a characteristic that could not be identified in IgG-SC from hospitalized children. Altogether our study suggests that, compared to subclinical (household) pediatric cases, the anti-sE IgG-SC response in hospitalized pediatric cases is similar in frequency among total IgG-SC, but that these sE-specific IgG-SC are significantly lower in numbers, weaker in affinity and their antigen specificity less broad.

Plasmablasts are only present in the blood during a short window after infection, however, they can contribute substantially to the overall antibody response. Investigation of this compartment for epitope specificity is challenging, and mostly B cell ELISPOTS have been used to determine the binding properties of IgG-SC, sometimes expanding to the analysis of cross-reactivity to two DENV serotypes or ZIKV using adapted FLUOROSPOT assays [59–62]. ELISPOT and FLUOROSPOT resemble the DropMap technique we used herein in that they consider antibody-secreting cells rather than cells identified by flow cytometry that express a given set of membrane-expressed markers that classifies them as ‘plasmablasts’. Previous reports described association of high frequencies of plasmablasts/plasma cells identified using flow cytometry or scRNAseq approaches with more severe dengue infections [34–36,55–57]. However, not all plasmablasts may secrete antibodies, similarly to myeloma or hybridoma cell lines that display 10–40% secretors at a given time in culture. An alternative, labor intensive approach is the cloning and expression of BCRs from single cell sorting of plasmablasts that bound fluorescently-labeled DENV proteins [63,64]. This approach will identify the antibody subclass, but does not test for secretion of that antibody during the cell sorting. Of note, these single-cell sorts have shown that the plasmablast repertoire (which serves as an approximation of the circulating antibody repertoire) constitutes only a small subset of the virus-specific memory B cell repertoire during post-primary infection. However, the FACS-based approach shows that only ~50% of the identified repertoire actually binds DENV proteins after antibody re-expression [37].

Efforts to describe the circulating antibody repertoire after DENV infection have mainly concentrated on the polyclonal plasma response for which single antibody reactivities and affinities cannot be evaluated or on the production of limited number of monoclonal antibodies [37–40,65,66]. Here we employed the DropMap technique [41] that interrogates the secreted IgG antibodies from IgG-SC for specificity and affinity towards a given antigen. For the first time, we adapted the assay to evaluate binding affinity and specificity to three antigens simultaneously in each droplet, here, 3 different DENV E-derived proteins. We included disulfide stabilized dimers with sequences derived from Cambodian patient isolates. To stabilize the sE dimers, 2 mutations were introduced to create 2 disulfide bonds, including one mutation in the fusion loop. Hence, this locked dimer does not expose the fusion loop [33]. Comparing the binding profiles of antibodies secreted by single IgG-SC for sE-dimer or monomers allowed us to discern the epitope specificity; IgG-SC with specificity only to monomeric sE but not sE-dimer secrete most probably antibodies targeting the FL, whereas IgG-SC with specificity only to sE-dimer but not sE-monomer secrete most probably antibodies targeting quaternary epitopes, and IgG-SC with specificity to both monomeric sE and sE-dimers secrete EDIII- or EDII-specific antibodies.

Rare sE-specific IgG-SC could be identified from healthy donor samples (<10%), whereas in dengue-infected cases, 30–100% of the IgG-SC recognized sE-derived antigens. IgG-SC recognizing sE-dimers from DENV1 and DENV2 from both subclinical and hospitalized patients displayed a higher affinity towards the sE-dimer compared to IgG-SC recognizing only 1 sE-dimer, either from DENV1 or from DENV2. This is probably due to the boosting effect of past infections, where memory B cells were recruited in GC reactions for additional affinity maturation. As the antigens used in the DropMap assays were limited to sE and E-dimers, no information on antigen specificity is available for the remaining IgG-SC that were found not specific for sE or E-dimer from DENV1 and DENV2. Most probably these secrete IgG specific for unrelated, non-DENV-derived, antigens, but with a fraction specific for other DENV-derived proteins, such as NS1 and prM, that could also contribute to either disease enhancement or protection [60,67,68].

No significant difference in secretion rate could be identified between IgG-SC from healthy donors, subclinical cases and hospitalized dengue patients, whether considering all IgG-SC or IgG-SC producing IgG binding at least one recombinant E protein from DENV1 or DENV2. However, the affinity of these latter IgG antibodies for the sE monomers and sE-dimers were in general 2–10-fold weaker than those found in samples from subclinical patients. This reduced affinity of the secreted anti-E IgG antibodies in hospitalized patients may lower their neutralization potential but increase their ability to induce ADE, as suggested earlier [69,70]. This is also reflected in the epitope specificity of the IgG-SC: hospitalized patients have more IgG-SC binding monomeric sE only, without binding the sE-dimer. As only the monomeric sE proteins expose the fusion loop, these IgG-SC probably secrete anti-fusion loop antibodies that have been reported poor neutralizers and that can mediate ADE in vitro and in vivo [21,26–28,30]. In subclinical cases, however, more IgG-SC are detected that are serotype-cross reactive, which might indicate that these individuals have a broader repertoire with anti-E antibodies binding to multiple serotypes that could protect them from disease. In addition, antibodies from IgG-SC recognizing both sE-dimers, but not binding to sE monomers, indicative of anti-quaternary epitope antibodies, were only detected in subclinical cases (i.e., in 5 out of 6 subclinical patients). Monoclonal antibodies of this class, such as C8 and C10 mAbs, bind with high affinity to the E-dimer epitope and neutralize all four DENV serotypes and ZIKV by preventing viral fusion, with demonstrated protective efficacy in murine models [31,71]. It remains to be determined what the relative contribution of pre-existing antibodies produced by long-lived plasma cells and newly generated antibodies originating from these circulating antibody-secreting cells are with respect to neutralization or disease enhancement during acute post-primary infection [72,73].

Our study has limitations. First, our cohort design excluded formal information on the serotype of past dengue infections that we inferred from the results of an EDIII binding assay on the acute plasma sample. Second, all our analyses had to be performed on frozen PBMC samples from pediatric cohorts, limiting the number of cells for DropMap analyses with the risk of reducing the proportion of detectable IgG secretion from cells after the freeze-thaw cycle disproportionally between patient groups. Third, inherently to the DropMap technology, the number of cells analyzed per experiment is comprised between 20,000 and 40,000 PBMCs which resulted in identification of ~300 IgG-SC per experiment for subclinical cases but only ~45 IgG-SC per experiment for hospitalized cases due to their reduced numbers of circulating IgG-SC. This limitation could partly be compensated by using statistical methods including patient effects. Low IgG-SC numbers in samples may explain the lack of detection of IgG-SC binding quaternary epitopes in hospitalized samples, even though mathematically we had one chance out of 230,000 to have missed these cells in all nine samples if one considers that their proportion should be similar to that in subclinical patients, i.e., 3% of IgG-SC. Lastly, we might have missed some IgG-SC binding to quaternary epitopes, as sE-monomers in solution may dimerize in the presence of antibodies binding to quaternary epitopes [33].

Taken together, our data obtained from the study of a Cambodian pediatric cohort suggests that humoral immune responses in subclinical cases during post-primary infection is characterized by the production of high-affinity and cross-reactive anti-sE IgG antibodies with a fraction of them binding to quaternary epitopes. This study highlights the importance of humoral response monitoring and antigen design for vaccine strategies as it reports on the subtle changes in circulating antibody responses that could not be identified using bulk analyses of patient’s serum and might orient towards protective or pathogenic outcomes of the anti-dengue humoral response.

## Methods

### Study approval

This study was conducted in compliance with the Declaration of Helsinki principles and was approved by the National Ethics Committee of Health Research of Cambodia (NECHR nr 2017-113, nr 2018-170). Written informed consent was obtained from all participants or the guardians of participants before inclusion in the study.

### Patient recruitment

Patients were included during the dengue seasons of 2018 and 2019. Dengue patients were recruited at admittance to the Kampong Cham provincial hospital. Blood samples were obtained from hospitalized children (≥2 years) who presented with dengue-like symptoms. Hospitalized patients were classified according to the World Health Organization (WHO) 2009 classification criteria into uncomplicated dengue, dengue with warning signs (DWWS) and severe dengue (SD) upon hospital discharge [5]. Subclinical dengue-infected patients were identified using a household investigation approach around the identified hospitalized dengue cases in Kampong Cham during the same dengue seasons as described previously [46,74]. A blood sample was requested from all household members and individuals living in a 200m radius from the house of hospitalized index cases. In case of laboratory-confirmed dengue infection, signs and symptoms of dengue were recorded 5 days retrospective and 10 days prospective with a visit at D3, D5 and D10 after inclusion. Subclinical infection was defined as no or mild (<38 °C) fever for a maximum of 2 consecutive days and a maximum of 1 of the following symptoms for a maximum of 2 consecutive days: nausea or vomiting, rash, aches and pains. All individuals showed at least one symptom. Individuals requiring medical care were excluded from subclinical cases. None of the subclinical cases met the WHO 2009 classification criteria for dengue. At inclusion (day of RT-PCR confirmed DENV diagnosis, D0), five days later (D5) and 10 days later (D10) a blood sample was taken for immunological analysis (see below). Hospitalized and subclinical cases were matched for days of onset of symptoms, ensuring that analysis were performed at similar timepoints in the disease dynamics. Patient characteristics of the included patients and healthy donor controls is summarized in Tables 1 and 2.

### Laboratory diagnosis

Plasma was separated by density gradient centrifugation and tested for the presence of DENV by real-time reverse transcription polymerase chain reaction (RT-qPCR) as described before [75]. The infecting DENV serotype was subsequently confirmed by serotype-specific PCR [75]. Patients were identified as having primary/post-primary DENV infection using a hemagglutination inhibition test in line with the WHO criteria using the D10 plasma sample [76]. Peripheral blood mononuclear cells (PBMCs) obtained from whole blood collected in Heparin tubes were isolated from human blood samples obtained at D5 by using Ficoll–Histopaque (Sigma-Aldrich) density gradient centrifugation. The cells were resuspended in 10% of dimethyl sulfoxide (Sigma-Aldrich) and 90% of fetal bovine serum (FBS) (Thermo Fisher Scientific) and stored in liquid nitrogen until phenotyping analysis, or shipped to Institut Pasteur, Paris, France for DropMap analysis.

### DENV protein production

DENV E recombinant proteins, soluble E (sE) (50 kDa) and stabilized sE-dimer (100 kDa) proteins were produced, based on sequences of Cambodian DENV1 (accession number, AHG23167) and DENV2 (accession number, AHG23166) isolates, in Drosophila S2 cells as previously described (30,40), with the addition of a His-tag, an Avid-tag and a double Strep tag at the C-terminus. Two mutations were introduced in the sE-protein sequence obtained from Cambodian DENV2 isolate (Leu107Cys and Ala313Cys) to generate two disulfide bonds which will keep the sE in a dimeric form using the same strategy described in [33].

### DENV protein labelling

DENV proteins were labelled using NHS ester dyes (AF555, FP-U90663, FluoProbes; DY-490 and DY-749P1, Dyomics), according to the manufacturers’ recommendations (overnight incubation on ice) and with a molar excess of 8, at dye concentrations of 5, 2 and 2 mg/mL, respectively. A purification step was then performed using a centrifugal unit (Millipore) with 4–5 rounds of centrifugation at 16,000 g for 10 minutes. In each assay, DY-490 and AF555 were used for the infected variant proteins, and DY-749P1 was used for the cross-reactive variant.

### Anti-DENV-E IgG detection via MIA

*Determination of sE and sE-dimer antibodies in plasma obtained at day 5 after RT-PCR confirmed DENV diagnosis:* Color-coded magnetic microspheres beads were coupled with optimized concentrations of DENV1 and DENV2 sE and sE-dimer. The protein-coupled beads were incubated with 100 µL of purified IgG isolated from patient’s plasma (using protein G Sepharose 4 Fast Flow, GE Healthcare) at a concentration of 1µg/mL in assay buffer (PBS1X, 2% FBS (Gibco), 1% bovine serum albumin, BSA (Sigma), and 0.1% Tween-20 (Bio-Rad)) for one hour at RT in the dark, on a plate shaker at 450 rpm.

*Determination of anti-EDIII antibodies in plasma obtained at time of RT-PCR confirmed DENV diagnosis:* Recombinant EDIII proteins (DENV1–4 and Zika) coupled beads (a kind gift from Jessica Vanhomwegen, Institut Pasteur Paris, [77] were used. The plasma samples were diluted in assay buffer (1:100) and 100µL of pre-diluted sample were incubated with EDIII-coupled beads for one hour at RT in the dark, on a plate shaker at 450 rpm.

Both assays were washed for five times with assay buffer (PBS1X containing 0.1% tween 20). Afterward, 0.1µg R-Phycoerythrin AffiniPure goat anti-human IgG (Fisher scientific) was added. The complexes were incubated again for one hour at RT in the dark, on a plate shaker at 450 rpm. Antigen-antibody reactions were analyzed with the BioPlex-200 system (BioRad). For each sample, the median fluorescence intensity (MFI) was calculated from at least 50 beads bearing the same antigen. The assay was standardized and dynamic range was determined using well described monoclonal antibodies as described in [78].

### Foci reduction neutralization test (FRNT)

The neutralizing antibody titer against infecting and cross-reactive serotype was determined by FRNT assay using plasma obtained at day 5 after RT-PCR confirmed DENV diagnosis. Following DENV reference strains were used: DENV1 (Hawaii) and DENV2 (New Guinea C). Briefly, Vero-CCL cells (ATCC CCL-81) were seeded into 96-well plates one day prior to the assay. Heat-inactivated plasma from patient was serially diluted in DMEM starting at a 1:20 dilution. The pre-diluted plasma was incubated with DENV1 (Hawaii) or DENV2 (New Guinea C) for 1 hour at 37°C, 5% CO2. Cells infected with the virus in the absence of plasma served as controls. Afterwards, Plasma-virus complexes were transferred onto 96-well culture plates containing the Vero CCL cells. Following 90 minutes incubation, the mixture was replaced with 1.8% carboxymethyl cellulose (Sigma-Aldrich) and incubated at 37°C, 5% CO2. At 2–3 days post-infection, the cells were fixed and stained as described [79]. The titer of neutralizing antibodies was expressed as the reciprocal plasma dilution that induces 50% reduction of infection, visualized as foci (FRNT50) compared to the controls well. The FRNT50 was calculated using non-linear regression analysis GraphPad Prism 10.3.1.

### Flow cytometry

The cryopreserved cells were thawed into warm RPMI (Sigma-Aldrich) added with 10% FBS and counted for flow cytometry analysis. After counting, 1*10^6 viable cells were used for the staining. First, the cells were stained with Zombie Aqua fixable viability kit (Biolegend) to determine live/dead cells. After washing, cells were stained for 30 min on ice with surface markers (CD19 APC-cy7, CD3 BUV395, IgM BV421, IgD BV711, IgG FITC, IgA APC, CD27 PercP-cy5.5, CD38BV605, all Biolegend) After washing, cells were resuspended in PBS, 1%BSA, 2mM EDTA. The cells were analyzed with BD FACSAria Fusion (BD Biosciences).

### Antibody expression and purification

The human mAbs used in this study (Table 2) were isolated, cloned and expressed as previously described [80]. Antibody VH and VL sequences were found in the ADImab database, i.e., ADI-24191, ADI-24194, ADI-24197, ADI-24219, ADI-24229, ADI-24269, ADI-24292 and ADI-24370, or in the literature, i.e., 1A1D-2 [49], 5AAM [50], 6FLC and 6FLA [48]. Briefly, pUC-19-based vectors containing the constant regions of human IgG1 heavy chain and of the human kappa or lambda light chain and the VH and VL, respectively, of the aforementioned mAbs were co-transfected at a ratio of 1:3 (H:K/L) into Expi293F cells (Thermo Fisher Scientific Inc.) using the ExpiFectamine 293 Transfection Kit (Thermo Fisher Scientific Inc.). Seven days post transfection, the cell supernatant was collected, filtered (0.22 μm) and incubated with Protein A-coated agarose beads (GE Life Sciences) for 2 h at RT. The antibodies were eluted using 50 mM sodium phosphate (pH 3.0) and the pH was immediately adjusted using 1 M Tris-HCl (pH 8.0). Following buffer exchange to PBS, the reactivity of each mAb was confirmed by ELISA before it was aliquoted, and stored at -80°C.

### Affinity determination of monoclonal antibodies

Bio-layer interferometry measurements were performed using anti-human IgG sensors in a OctetRED system (ForteBio). Biosensors were equilibrated in assay buffer (PBS with 0,05% Tween 20 diluted in sterile water + 0,1% BSA) for at least 10 min prior to measurement. DENV proteins were captured on the sensors for 10 min, followed by a baseline in assay buffer (30 sec). Equilibrium dissociation constants (K_D_) were determined by monitoring over time the association between the immobilized protein and the mAbs in solution in duplicate for 7 increasing concentrations of each mAb. An irrelevant IgG mAb was used as negative control to subtract the background signal. Data analysis was performed using the Octet Analysis software (ForteBio), and K_D_ values were calculated by steady-state analysis. Traces were preprocessed by reference sample subtraction, alignment to the baseline average, and Savitzky-Golay filtering. Equilibrium dissociation constant (K_D_) was determined using a 1:1 binding model with global fitting in HT Analysis 11.1 software (ForteBio).

### DropMap technology

#### Aqueous phase I: Preparation of cells for droplet compartmentalization.

PBMC samples obtained at 5 days after RT-PCR confirmed infection, corresponding to day 7–8 after onset of symptoms were analyzed. The frozen PBMC samples were thawed in a 37°C water bath and diluted with warmed RPMI-GlutaMAX supplemented with 10% FBS and 3 U/mL DNase I (New England Biolabs). The samples were washed twice in 10% FBS-supplemented RPMI-GlutaMAX by centrifugation at RT at 350 g for 5 min. The PBMC were transferred into a 25 cm² flask and incubated at 37 °C and 5% CO_2_ for 1 h. After incubation, the cells were counted using a KOVA slide, after which they were diluted to a concentration of 10⁷ cells/mL in Dropmap buffer consisting of Fluorobrite DMEM (A1896701), 0.1% Pluronic F68 (24040032), 20 mM HEPES (15630056), 5% KnockOut Serum Replacement (10828028), 2 mM L-glutamine (25030024) and 50 U/mL penicillin/streptomycin (15070063) (all from Thermo Fisher Scientific), and 0.5% human serum albumin (Sigma-Aldrich, A9731).

#### Aqueous phase II: Preparation of beads and bioassay reagents.

Paramagnetic nanoparticles (Strep Plus 300 nM, Ademtech) were washed with Dulbecco’s phosphate-buffered saline with calcium and magnesium (DPBS++, ThermoFisher). Nanoparticles were resuspended in DPBS++ containing 1 μM biotin-labeled llama VHH nanobody that specifically binds the CH1 domain of all four human IgG subclasses (anti-hIgG-CH1, CaptureSelect; Thermo Fisher, 7103202100), and incubated 20 min at RT. After another wash with DPBS++, nanoparticles were resuspended in 5% pluronic F68 solution (24040032; ThermoFisher) and incubated 20 min at RT. The nanoparticles were washed again and resuspended in DropMap buffer containing fluorescent reporter proteins for a final concentration of 1.25 mg/mL beads. Reporter proteins included: AlexaFluor647-labeled F(ab’)_2_ fragment of rabbit anti-human IgG Fc-specific (ref 309-606-008, Jackson ImmunoResearch) used at 75 nM final in-droplet concentration; fluorophore-conjugated DENV E proteins used at 30 nM final in-droplet concentration, i.e., for the “infecting mix” AF555-labeled sE protein from DENV infecting serotype, DY490-labeled sE-dimer protein from DENV infecting serotype and DY749P1-labeled sE-dimer protein from DENV cross-serotype, or for the “non-infecting mix” AF555-labeled sE protein from DENV cross-serotype, DY490-labeled sE-dimer protein from DENV cross-serotype and DY749P1-labeled E-Dimer protein from DENV infecting serotype.

#### Droplet production and collection.

Droplets were generated using hydrodynamic flow-focusing on a microfluidic chip as described (2). The wafer master of SU-8 photoresist layer (MicroChem) with ~36 µm of thickness were manufactured using soft-lithography (8) and microfluidic chips were fabricated using soft-lithography in poly-dimethylsiloxane (PDMS; Sylgard) (2). The continuous phase comprised 2% (wt/wt) “008 Fluorosurfactant” (RAN Biotechnologies) in Novec HFE7500 fluorinated oil (3M). Aqueous phases I and II were co-flowed and partitioned into droplets. The flow rate of aqueous phases I and II was 70 µL/h, with an oil flow rate of 550 µL/h to achieve monodispersed droplets of ~40 pL volume. Newly generated droplets were directly injected into the DropMap 2D chamber system (2), mounted on a widefield fluorescence microscope (Ti2-Eclipse, Nikon). The emulsion was exposed to a magnetic field, forcing the nanoparticles inside each droplet to form an elongated aggregate termed “beadline”.

#### Data acquisition.

Images were acquired using a Nikon inverted microscope with a motorized stage (Ti2-Eclipse). Excitation light was provided by a light-emitting diode (LED) source (SOLAlight source, Lumencor Inc.). Fluorescence for the specific channels were recorded using appropriate band-pass filters and camera settings (Orca Flash 4.0, Hamamatsu) at room temperature and ambient oxygen concentration. Images were acquired using a 10x objective (NA 0.45). An array of 20,000x16,000 pixels images was acquired (covering ~80,000 droplets), every 7.5 min in all channels over 37.5 min (6 measurements total). Each chamber (chip) contains droplets corresponding to a single sample. Duplicates were acquired for every sample that had enough cells, with each replicate being the filling of the DropMap 2D chamber with a novel droplet population acquired over time on a 20,000x16,000 pixels image array.

#### Image analysis and calculations.

Image analyses consists of five stages: i) identifying each droplet and its spatial coordinates within the DropMap chamber; ii) identifying the beadline within each droplet; iii) excluding droplets containing no cell or an aggregate of cells; iv) extracting for each timepoint the fluorescence signals of the beadline, and of the entire droplet without the beadline; and v) calculating the fluorescence accumulation in each of the 4 channels on the beadline over time. Each droplet is assessed individually, both computationally and visually to ensure proper assay formation and data fidelity.

Images were analyzed using a custom-made Matlab script (Mathworks) to identify each droplet and its beadline. In each fluorescence channel and for each droplet, the pixel intensities of the beadline and the mean pixel intensities except the beadline (background fluorescence) were extracted. Droplet fluorescence relocation at each timepoint was calculated by dividing the intensity of the beadline by that of the background. Data was exported to Excel (Microsoft) and sorted on a home-made Python software for droplets showing an increase in relocation of the anti-IgG reporter fluorescence (Alexa647) over time (i.e., Alexa647-relocation > 1.15) and others parameters like the difference in the fluorescent signal between the first and last points of acquisition (>0.03) or the value of the slope of the fluorescent signal vs time (>0.004) and its fitting parameter to a line (R² > 0.75). Sorted droplets were visually assessed for: the presence of a single cell within the droplet, no droplet movement between image acquisitions, the absence of fluorescent particles other than the beadline (e.g., fluorescent protein aggregates, cell debris), and undesired aggregation of fluorescent reporters on the cell surface inside the droplet. IgG secretion rate and dissociation constant (K_D_) were estimated as described (2).

#### Estimation of IgG concentration within each droplet.

*A* calibration curve for the estimation of IgG concentration was obtained by preparing droplet populations containing all bioassay reagents except cells that were replaced by a range of concentrations of monoclonal human IgG1 (one concentration per droplet population). Images were acquired exactly 3min after the droplets were immobilized in the DropMap chamber for each IgG concentration, and fluorescent relocation calculated. The calibration curve was subsequently used to estimate the IgG concentration and secretion rate (IgG molecules per second) for each time interval, and the mean secretion rate was calculated by averaging the values of all intervals.

#### Estimation of dissociation constant (K_D_) for DENV proteins within each droplet.

In standard DropMap assays, a single fluorescently labeled antigen is present in a droplet. However, in this study, three similar antigens are present in each droplet, either sE-dimer (DENV1), sE-dimer (DENV2) and sE (DENV1), or sE-dimer (DENV2), sE-dimer (DENV1) and sE (DENV2). These DENV proteins are thus in competition for the binding of an anti-E protein IgG antibody secreted by an IgG-SC within the droplet. We therefore established reference curves for each case: an antibody leading to the relocation of 1, of 2 or of 3 different sE-proteins. Calibration curves were obtained by defining relocation values for both anti-IgG (AF647) and the antigen for a collection of 3–6 monoclonal mAbs with different K_D_ for that antigen. Droplet populations were generated for different concentrations of each mAb to be analyzed by the DropMap bioassay and for each antigen binding conditions, whether binding 2 or 3 antigens. A curve was defined by plotting the relocation from the anti-IgG (AF647) against relocation of the antigen, and the slope of the resulting line was calculated and termed “DropMap slope”. The calibration curve was defined by the power relation between the K_D_ of the mAb for the antigen and the DropMap slope of each mAb. This allows for the estimation of a K_D_ value for each droplet containing an unknown anti-E protein IgG secreted by an IgG-SC as it defined a DropMap slope value (refer to S3 Fig).

### Statistics

Data were neither averaged nor normalized prior to analyses. All points present single cell values unless otherwise stated. Each PBMC sample was measured in 2 independent DropMap experiments when the sample size allowed it, and data points were pooled between duplicate experiments.

GraphPad Prism v9.4.1 or the R environment v4.6.0 [81] were used for all the analyses. Response variables were log2 converted for better adjustment to linear models. Data were fitted to a linear model that includes the variables of interest and some random effect: Fig 2, clinical status (hospitalized or subclinical) and random effect of patients; S2 Fig, random effect of patients; S4 Fig, protein bound, patient and random effect of cells; Fig 3, protein bound and random effect of patients and cells; S7A Fig, protein bound and random effect of patient. Age and sex covariates were not incorporated, as their effects were already represented by the patient variable. Mixed models using the lmer() function of the lme4 package were used to define the random effects. ANOVAs were performed with the Anova() function of the car package. Type 3 sum of squares was applied due to unbalanced designs. Two-by-two effect comparisons (contrast comparisons) were performed with the emmeans() function of the emmeans package. Statistical significance was set to a P value of 0.05 or less. In each panel, type I error was controlled by correcting the P values according to the Benjamini & Hochberg method [“BH” option in the p.adjust() function of R]. For Figs 4A, S5 and S6, Kruskal-Wallis test with Dunn’s multiple comparisons’ test were used, and for S1D Fig, a Mann-Whitney test was performed.

## Supporting information

S1 Fig (A) Schematic representation of the DropMap workflow. PBMC from hospitalized and subclinical dengue cases were collected at 7–8 days after onset of symptoms and frozen. After thawing, IgG-SC within each PBMC sample were analyzed by DropMap. Created in Biorender. **(B)** IgG secreting rate from (left) total IgG-SC or (right) IgG-SC binding at least one sE or sE-dimer of DENV1 or DENV2 from dengue infected cases (cumulative data of hospitalized and subclinical cases; n = 15) and healthy donors (HD; n = 3). The number of DropMap experiments (assays) performed for each group is indicated. Horizontal bars represent median and interquartile range. Each dot represents one IgG-SC. **(C).** Gating strategy for the identification of CD19^+^ IgD^-^ IgM^-^ IgG^+^ cells and CD19^+^ IgD^-^ CD27^+^ CD38^++^ ASC. **(D)** Percentages of CD19^+^ IgD^-^ IgM^-^ IgG^+^ cells and CD19^+^ IgD^-^ CD27^+^ CD38^++^ plasmablasts in subclinical (blue) and hospitalized patients (red) at day 5 post RT-qPCR confirmed DENV infection, corresponding to day 7–8 after onset of symptoms. Horizontal bars represent median and IQR. *p* values are calculated using Mann-Whitney U test; ns, not significant; *, p ≤ 0.05.
(TIFF)

S2 Fig (A-B) IgG secreting rate from (A) total IgG-SC or from (B) IgG-SC binding to sE and/or sE-dimer of DENV1 or DENV2 in PBMCs from indicated hospitalized dengue patients and subclinical cases at day 5 post RT-qPCR confirmed DENV infection, corresponding to day 7–8 after onset of symptoms. Horizontal bars represent median and interquartile range. Each dot represents one IgG-SC. p values were calculated using two-by-two contrast comparisons after linear modelling. *, p ≤ 0.05; **, p ≤ 0.01; ***, p ≤ 0.001; ****, p ≤ 0.0001. Of note, non-significant differences are not represented.
(TIFF)

S3 Fig Affinity reference curves used for K_D_ calculations from DropMap data. Representation of DropMap slope vs KD data obtained using Biolayer Interferometry (Octet) of a series of anti-E protein monoclonal antibodies binding: **(A)** DENV1 sE and DENV1 sE-dimer, **(B)** DENV1 sE, DENV1 sE-dimer and DENV2 sE-dimer, **(C)** DENV2 sE and DENV2 sE-dimer, **(D)** DENV2 sE, DENV2 sE-dimer and DENV1 sE-dimer. **(A-D)** Power regressions and their formula are represented for each antigen relocation within a condition (i.e., if the antibody binds two or three different antigens, and from which dengue serotype). Each dot represents the mean of the measures for one anti-sE-protein mAb performed in triplicate; SEM are represented. Note: The competition between cross-reactive mAbs binding 2 or 3 different E antigens was addressed by calibrating the assay under the same multiplex conditions used for patient-derived IgG-secreting cells (Figs 2–4 and S4–S8). Here, we generated separate KD–DropMap slope relationships for the relevant binding configurations, including droplets in which two or three antigens were relocated. These curves were established using monoclonal anti-DENV antibodies with independently determined KD values, measured with the same antigen combinations and assay geometry.
(TIFF)

S4 Fig Dengue infected patients display IgG-SC of higher affinity for DENV1 sE-dimers than DENV2 sE-dimers irrespective of the infecting serotype. **(A-B)** DropMap-measured affinity (KD) of IgG-SC from indicated **(A)** subclinical or **(B)** hospitalized patients infected with DENV1. **(C-D)** DropMap-measured affinity (KD) of IgG-SC from individual **(C)** subclinical or **(D)** hospitalized patients infected with DENV2. **(A-D)** Data are divided by categories of IgG-SC that bind one sE-dimer or both sE-dimers, as indicated in the legend below the graphs. Horizontal bars indicate median and interquartile range. Each dot represents the KD value of one IgG-SC recognizing at least one sE-dimer. p values were calculated using two-by-two contrast comparisons after linear modelling. ns, not significant; **, p ≤ 0.01; ***, p ≤ 0.001; ****, p ≤ 0.0001. The dotted line indicates the 30nM affinity limit that discriminates E protein binders from non-binders. N.B.: non-binding.
(TIFF)

S5 Fig Comparison between the infecting assay and cross assay. DropMap-measured affinity (K_D_) of IgG-SC from subclinical patients infected with **(A)** DENV1 or **(B)** DENV2. **(A-B)** Data are divided by categories of IgG-SC that bind one sE-dimer or both sE-dimers, and the nature of the DropMap assay, i.e., “infecting” or “cross”, as indicated in the legend. Horizontal bars represent median and interquartile range. Each dot represents one IgG-SC recognizing at least one sE-dimer. The dotted line indicates the 30nM affinity limit that discriminates E protein binders from non-binders. *p* values are calculated using Kruskall-Wallis followed by with Dunn’s multiple comparison test. ns, not significant; *, p ≤ 0.05; **, p ≤ 0.01; ****, p ≤ 0.0001. N.B.: non-binding. *Note*: The “infecting” assay contains sE and sE-dimer antigens of the serotype infecting the patient analyzed, and the sE-dimer of the other serotype (i.e., non-infecting). The “cross” assay contains the sE-dimer of the serotype infecting the patient analyzed, and the sE and sE-dimer of the other serotype (i.e., non-infecting).
(TIFF)

S6 Fig Comparison of affinity of anti-sE/sE-dimer antibodies in healthy donors and DENV-infected patients. DropMap-measured affinity (K_D_) of IgG-SC from Cambodian age-matched healthy pediatric donors (HD) and Cambodian subclinical and hospitalized pediatric patients infected with **(A)** DENV1 or **(B)** DENV2. **(A-B)** Horizontal bars represent median and interquartile range. Each dot represents one IgG-SC recognizing at least one sE-dimer. The dotted line indicates the 30nM affinity limit that discriminates E protein binders from non-binders. *p* values are calculated using Kruskall-Wallis followed by Dunn’s multiple comparison test. ns, not significant; *, p ≤ 0.05; **, p ≤ 0.01; ****, p ≤ 0.0001.
(TIFF)

S7 Fig IgG-SC affinities for DENV sE and sE-dimers from individual subclinical and hospitalized patients. DropMap-measured affinity (K_D_) of sE-protein-specific IgG-SC binding in subclinical or hospitalized patients infected with (left) DENV1 or (right) DENV2. Horizontal bars indicate median and interquartile range. Each dot represents one sE-protein-specific IgG-SC. *p* values were calculated using **(A)** Kruskall-Wallis followed by Dunn’s multiple comparison test to compare subclinical and hospitalized samples, or **(B)** two-by-two contrast comparisons after linear modelling to compare samples within a patient group. ns, not significant; *, p ≤ 0.05; **, p ≤ 0.01; ***, p ≤ 0.001; ****, p ≤ 0.0001. The dotted line indicates the 30nM affinity limit that discriminates E protein binders from non-binders.
(TIFF)

S8 Fig Proportions of IgG-SC binding to the different sE and/or sE-dimer proteins in both patient groups infected with DENV1 (left) or DENV2 (right). Patient ID (S# or H#), number of patients (n), total number of anti-E protein IgG-SC analyzed and percentages of anti-E protein IgG-SC falling in each category are indicated.
(TIFF)

S1 Data Raw data for Figs 1–4. (ZIP)

S2 Data Raw data for S1–S8 Figs. (ZIP)

S1 File Publication license. (PDF)
