# Supplementary material for: Quantitative and qualitative differences in anti-Envelope IgG-secreting cells distinguish subclinical and hospitalized dengue in a Cambodian pediatric cohort
Source: PLoS Pathog. 2026 Oct 1;22(10):e1014552. doi: 10.1371/journal.ppat.1014552 (PMC13649127; doi:10.1371/journal.ppat.1014552)
Supplement: Supplementary file 2 [file ppat.1014552.s013.docx]

**Point by point reply to the Reviewer's suggestions**

**Part I – Summary**

Please use this section to discuss strengths/weaknesses of study, novelty/significance, general execution and scholarship.

**Reviewer #1**: This an interesting study investigating how antibody responses differ between subclinical and hospitalized dengue infections, focusing on IgG-secreting cells (IgG-SC) at the single-cell level. Using a droplet-based microfluidic platform, the authors analyze over 3,300 individual cells from a pediatric cohort to measure antibody secretion, specificity, affinity, and cross-reactivity toward dengue envelope (E) protein antigens. They report that while hospitalized patients exhibit a higher proportion of dengue antibodies, these antibodies are of significantly lower affinity to the dimeric quaternary form of E protein compared to those from subclinical cases. Whereas hospitalized patients predominantly produce monomer-reactive antibodies, which were previously linked to poor neutralization and antibody enhancement of infection. The findings offer an important conceptual advance by linking disease severity not to the magnitude of the antibody response, but to its qualitative features; specifically, affinity and epitope targeting. The observation that subclinical cases harbor rare but potentially protective quaternary epitope–specific antibodies provide support for vaccine strategies that aim to elicit such responses. The study also reinforces the idea that high-frequency, low-affinity, and monomer-directed responses may contribute to disease pathology.

**Reviewer #2**: Bruhns and coworkers used the very interesting and powerful DropMap technology they had developed earlier for the single cell level analysis of antibody secretion and affinity to characterize the response to subclinical or hospitalized post‑primary dengue (DENV1 or DENV2) in a pediatric cohort. The key finding is that hospitalized patients show a higher fraction of DENV‑specific ASCs which however produce lower‑affinity antibodies, are skewed toward monomer (sE) binding but not towards protective quaternary epitopes. The work is technically innovative, conceptually interesting and the data intriguing.

We thank the Reviewers #1 and #2 for their positive view on our work. We have addressed all the issues and concerns raised and provide below a detailed point-by-point reply.

**Part II – Major Issues: Key Experiments Required for Acceptance**
Please use this section to detail the key new experiments or modifications of existing experiments that should be absolutely required to validate study conclusions.

**Reviewer #1**: 1. A substantial number of cells were analyzed in this study, with results presented as pooled data across all cells and all donors in the same group. However, given the relatively modest cohort size (6 and 9 donors), pooling across individuals may introduce bias. This is particularly relevant if certain donors contribute disproportionately larger numbers of cells, potentially skewing the overall trends. The authors acknowledge this limitation in the Discussion, noting that fewer cells were obtained from hospitalized samples compared to subclinical. Nonetheless, to ensure that the observed reported patterns and differences between groups are not driven by only a small subset of individuals it is important to provide a clear breakdown of the number of cells analyzed per donor. If substantial cell number differences exist between individuals, this needs to be addressed and if this is the case, normalization or down-sampling approaches should be considered to reduce overrepresentation from donors contributing more cells. More broadly, key comparisons including IgG secretion, antigen binding, frequency of positive cells, affinity, and epitope specificity, should be also analyzed at the individual donor level to strengthen the confidence of the main findings and ensure that the findings are representative of the cohort as a whole and not driven by only a few donors withing each group.

We thank the reviewer for suggesting individual donor level analyses. To properly address this concern, we associated a biostatistician, Dr Gaël A. Millot (now co-author) to the revision phase of this manuscript. We provide now:

- Re-analysis including patient effect on IgG secretion (Figure 2B): the small (1.5-fold difference) was explained by a patient effect. Individual patient data and analyses for IgG secretion are now presented as novel Supplementary Figure 2 to complete the bulk analysis represented in Figure 2B. We modified our conclusions in the main text.
- Individual patient analyses for affinity of IgG secreted by IgG-SC towards DENV1 sE dimer and/or DENV2 sE dimer (novel Supplementary Figure 4) to complete the bulk analysis represented in Figure 3. Figure 3 was reanalyzed to include potential patient effects, but differences we observed initially remained true.
- Individual patient analyses for affinity of IgG secreted by IgG-SC towards DENV1 sE dimer, DENV1-sE, DENV2 sE dimer and DENV2-sE (new Supplementary Figure 7) to complete the bulk analysis represented in Figure 4A. Due to the high number of comparisons to provide, Supplementary Figure 7 has two panels representing the same data, but providing different analyses: panel A compares subclinical with hospitalized groups, and panel B compares individuals within each group.
- Individual patient analyses for epitope specificity of IgG secreted by IgG-SC (new Supplementary Figure 8) to complete the bulk analysis represented in Figure 4B.

For all individual data, the number of IgG-SC analyzed is indicated in the new and revised Figures. Statistical differences are indicated between patients falling into the same group in each new Figure to highlight – in general – the absence of significant variations between patients, particularly regarding affinity for DENV E proteins.

Data are now analyzed differently than in the first submission. It was neither averaged nor normalized prior to analyses. Response variables were log2 converted for better adjustment to linear models. Data were fitted to a linear model that includes the variables of interest and some random effect, including (when it applies): clinical status (hospitalized or subclinical), random effect of patients, protein bound, patient and random effect of cells. Age and sex covariates were not incorporated, as their effects were already represented by the patient variable. Whenever possible, two-by-two effect comparisons (contrast comparisons) were performed and corrected according to the Benjamini & Hochberg method. For the remaining analyses, Kruskal-Wallis test with Dunn’s multiple comparisons’ test or a Mann-Whitney test were used.

Overall, our conclusions remained the same as in the initial submission (except for IgG secretion that was not significant when including the patient effect). Individual data provided in Supplementary Figures 2, 4, 7 and 8 showcase inter-patient variations in this small cohort with, nevertheless, conserved differences (or trends) compared to the pooled data.

**Reviewer #2**: A few points need to be addressed before publication:

1. The distinction between “subclinical” and “hospitalized” dengue is central to the paper but not well defined. How many subclinical cases were completely asymptomatic vs mildly symptomatic? Did any meet WHO “dengue” criteria but did not require hospitalization?

We thank the reviewer for the comment. None of the “subclinical” patients met the WHO 2009 criteria for dengue, as subclinical infection was defined as no or mild (<38 °C) fever for a maximum of 2 consecutive days and a maximum of 1 of the following symptoms for a maximum of 2 consecutive days: nausea or vomiting, rash, aches and pains. All "subclinical” patients experienced at least one symptom, as patients were matched in the disease course based on the onset of symptoms. This information has been added to the materials and methods section and results section of the manuscript

2. Cell numbers in hospitalized patients are lower. To what extent this factor could have influenced the key conclusions here? Also the effect of freeze thaw on plasma blast viability (which may be affected) by disease status should be at least discussed.

We have indicated the numbers of IgG-SC analyzed for each patient in the new supplemental Figures #2, 4, 7 and 8. As we stated in the first submission, the number of IgG-SC analyzed was significantly (10-fold) lower in hospitalized patients (483 IgG-SC analyzed for 9 patients) compared to subclinical patients (2498 IgG-SC analyzed for 6 patients). On an individual basis (refer to new Supp. Fig. 2), this difference maintains except for subclinical patient c0628658 (only 41 IgG-Sc analyzed) and hospitalized patient c0822507 (88 IgG-Sc analyzed). As pointed out by this Reviewer, and as requested by Reviewer#1, we present individual data in the revised supplemental Figures #2, 4, 7 and 8, and performed statistical comparisons including potential patient effects to report on intra-group variations. The difference in IgG-Sc numbers analyzed per patient is included in the novel statistical analyses using the two-by-two effect comparisons (contrast comparisons) described in the Material and Methods section, and is reflected in the *p* values provided. Overall, our conclusions remained the same as in the initial submission (except for IgG secretion that was not significant when including the patient effect).

Samples were collected in Cambodia, PBMC isolated and frozen once for shipping to France. No DropMap analysis could be performed in Cambodia on fresh (non-frozen) samples to evaluate the effect of a freeze/thaw cycle. We reported however earlier, investigating anti-SARS-CoV-2 RBD IgG-SC in vaccinated patients that the distribution of affinities was conserved between fresh and frozen samples, with 20-30% less IgG-SC detectable after freeze/thaw cycle (Broketa, M. et al. Qualitative monitoring of SARS-CoV-2 mRNA vaccination in humans using droplet microfluidics, JCI Insight 8 (2023). <https://www.ncbi.nlm.nih.gov/pubmed/37252802>). We therefore do not expect a change in affinity and specificity distribution among IgG-Sc, but a reduction in viable IgG-SC of 20-30% in all samples, whether from subclinical or hospitalized patients. This limitation is mentioned in the “limitations” paragraph at the end of the discussion.

3. The findings regarding higher frequencies of ASCs producing sE specific antibodies as opposed to quaternary epitopes in hospitalized patients is extremely interesting and probably the most significant finding from this work. But then neither neutralization nor binding titers were different between the two groups. Perhaps a per patient correlation analysis of FRNT/MIA and DropMap parameters could be illuminating. Separately could it be that FRNT/MIA are strongly influenced by pre-existing circulating antibodies from LLPCs which would not be detected here? In any event at the very least this issue needs to be addressed in the Discussion.

Indeed, neutralization titers or binding titers do not differ in our study between subclinical and hospitalized patients. This could be attributed to the small size of our cohorts, and/or to the absence of correlation between neutralization potency or binding titers with protection, as reported earlier (DOI: [10.1038/s41467-024-53916-9](https://doi.org/10.1038/s41467-024-53916-9); DOI: [10.1056/NEJMoa1800820](https://doi.org/10.1056/nejmoa1800820)). Given the small cohort size and limited neutralization data available herein, a correlation analysis would not be meaningful. It remains indeed an intriguing question in the field exactly what the relative contribution of pre-existing antibodies and newly generated antibodies are with respect to neutralization. We have added this notion to the discussion of the manuscript.

We also provide in this revised version of the manuscript Individual patient analyses for epitope specificity of IgG secreted by IgG-SC (new Supplementary Figure 8) to complete the bulk analysis represented in Figure 4B. It highlights that 5 out of 6 subclinical patient samples contained quaternary epitope-binding IgG-SC whereas no hospitalized sample did.

4. The use of three competing E‑derived antigens per droplet is a very important advance for DropMap. Please explain more clearly how completion influences the measured slope and KD values especially for cross reactive EDIII antibodies.

We thank the reviewer for raising this important point. The DropMap slope is used here as an empirical affinity-dependent readout, rather than as a direct analytical measurement of KD. In droplets containing several E-derived antigens, a cross-reactive IgG may bind more than one antigen, such that the relocation signal measured in each antigen channel reflects both binding to that antigen and competition with the other antigens present in the same droplet. In practical terms, competition may decrease the relocation slope observed in each antigen channel compared with a single-antigen assay, because part of the antibody-binding capacity is occupied by the other E-derived antigens; consequently, slopes are interpreted only within the corresponding multiplex calibration regime and not compared directly across relocation configurations.

This competition was addressed by calibrating the assay under the same multiplex conditions used for patient-derived IgG-secreting cells. As shown in Supplementary Fig. 2, we generated separate KD–DropMap slope relationships for the relevant binding configurations, including droplets in which two or three antigens were relocated. These curves were established using monoclonal anti-DENV antibodies with independently determined KD values, measured with the same antigen combinations and assay geometry. Importantly, Table 2 shows that several cross-reactive calibration mAbs displayed comparable affinities across the E-derived antigens tested, limiting the risk that the calibration curves were driven only by strongly unbalanced competition toward a single antigen.

Thus, the calibration does not assume that antigens behave independently in the droplet. Instead, the effect of antigen competition on the slope–KD relationship is empirically captured within each measured multiplex configuration. Once the relocation pattern of an unknown antibody was assigned, the corresponding reference curve was used to estimate its affinity. We therefore interpret the reported values as calibrated apparent KD estimates within the multiplex DropMap assay, rather than as single-antigen solution-phase KD measurement.

We revised the legend of Supplementary Fig.3 (reference curves) to summarize this concept, and added a sentence in the result section to improve the clarity of our approach and calculation.

**Part III – Minor Issues: Editorial and Data Presentation Modifications**
Please use this section for editorial suggestions as well as relatively minor modifications of existing data that would enhance clarity.

Reviewer #1: Minor comments:
2. Line 120:” Binding to anti-EDIII from DENV1 tended to be higher than the other serotypes, irrespective of the current infecting serotype, except for one individual with a strong EDIII DENV-4 binding preference (Figure 1B)”- this trend is not readily apparent in the current presentation of the figure. For example, samples S1, H2, and H3 appear to show comparable reactivity across all DENV antigens tested, making it difficult to discern the stated pattern. More broadly, panels B and C are challenging to interpret in their current format. It may be helpful to reorganize these panels to improve clarity (presenting the data as a heatmap could make relative differences and trends more immediately apparent). As it stands, extracting clear conclusions from these panels is not straightforward. Importantly, the authors need to include the appropriate negative control antigens to distinguish polyreactive serum responses from those that are specific to DENV or ZIKV.

We have added a heatmap to the panel B to visualize the MFI differences between the serotypes. We agree with the Reviewer that sometimes the differences are subtle and might not be meaningful (eg. patient S1, H2, H3, H5, H9), while for other patients the difference is more pronounced (eg. H1, S3, H7, S4, H8, S5, S6, H6). We have modified the text in the results section accordingly.

3. In Figure 2, it is not clear whether the sE/sE-dimer positive IgG SCs shown in dark green indicate cells that bind both forms, only one form (and which one) or either one of the forms? Again, data representation can be improved.

We apologize for the lack of clarity in the Fig.2C and its legend. We modified the legend in the Figure and the text of the legend. We added different colored boxes to make the figure more understandable.

4. The observation that approximately 10% of IgG-secreting cells from healthy donors show binding to DENV1/2 is somewhat unexpected and would benefit from further clarification. Could the authors comment on the nature of these responses? For example, do these cells represent low-affinity or polyreactive antibodies, or might this reflect background signal inherent to the assay? Related to this, it would be helpful to better understand how binding was defined. If this signal represents baseline assay noise, should a more stringent threshold be applied to classify an IgG-secreting cell as “binding”? Providing additional detail on how the signal was measured, including the criteria and thresholds used to define positivity, would greatly improve interpretability. Overall, further clarification of these points would help distinguish true antigen-specific responses from background reactivity.

The DropMap assay was setup using irrelevant IgG mAbs and a collection of DENV-specific mAbs reported by Adimab or in the literature. The threshold for defining “binding” was set at 30nM to avoid low-affinity polyreactive mAbs to be falsely included. The healthy donors were aged-matched Cambodian children that may have been exposed to viruses with similarities in their envelope proteins to DENV envelope proteins, and therefore possess IgG-SC raised against other viruses but with cross-reactivity to DENV. The few IgG-SC of the Healthy donors binding to the sE-derived antigens showed indeed a low binding affinity compared to DENV-infected patients (Supplementary Figure 6).

5. It seems that the IgG secretion rate in total IgG is not different from the secretion rate in DENV binding IgG. Is it possible that these differences are patient specific/intrinsic and have no relation to the infection severity?

Re-analysis including patient effect on IgG secretion (Figure 2B) demonstrated that the small (1.5-fold difference) we initial reported was explained by a patient effect. Individual patient data and analyses for IgG secretion are now presented as novel Supplementary Figure 2 to complete the bulk analysis represented in Figure 2B. We modified our conclusions in the main text to state that the secretion rate is not significantly different between patient groups.

6. Line 177: “The percentage of IgG-SC binding to at least one DENV sE protein among all IgG-SC was significantly lower in subclinical cases compared to hospitalized patients infected with DENV1 (41% vs 77%, respectively; 1.8-fold difference) and DENV2 (49 vs 61%, respectively; 1.2-fold difference) (Figure 2C)”. Can the authors explain what is the meaning of this? In general, it would be helpful to add a short conclusion sentence at the end of each Results section to increase clarity of the text.

This result indicates that the proportion of anti-E protein IgG-SC within all IgG-SC analyzed was significantly higher in hospitalized patients than in subclinical cases. We have modified the language in the result section.

For clarity, we have added title subheadings in the results section and a conclusion sentence at the end of each results section.

7. The error bar in Figure 4A looks off set.

We apologize for this oversight. We corrected the placement of the error bars within the new statistical analyses.

8. Why affinities in the hospitalized cases were not analyzed?

As the detectable number of anti-sE protein IgG-SC in hospitalized patients was low in DENV1-infected (# of IgG-SC from 0 to 11) and DENV2-infected (# of IgG-SC from 0 to 10), we initially decided not to display these results. Following the Reviewers request, we called upon a biostatistician to help with analyses and provide statistical methods that incorporated low cell numbers in the patient effect. We display them now as pooled data in New Figure 3B and 3D, and for each patient in New Supplementary Figure 4B and 4D. We performed statistical comparisons including the patient effect demonstrating better affinity for DENV1-SE dimer for IgG-SC specific for both DENV1 and DENV2 sE dimers compared to IgG-SC specific only for DENV1-sE dimer (Figure 3B; affinity for DENV1 sE-dimers, green dots) and (Figure 3D; affinity for DENV1 sE-dimers, green dots, and affinity for DENV2 sE-dimers, blue dots).

9. Figure 4B: representation could be improved to increase clarity. Number of subjects and number of cells need to be indicated. The method for statistical analysis needs to be indicated.

We apologize for the lack of clarity of Figure 4B in the first version of the manuscript. We have chosen pie chart representations including all percentages indicated per pie slice, cell numbers, and number of patients. The statistical method initially used was considered inadequate as some categories are not populated in some samples, and stats have been removed.

**Reviewer #2**: Minor: The authors used disulfide stabilized dimers however these can limit access to epitopes such as fusion loop compared to non-covalent dimers. This possible limitation should be mentioned in the Discussion.

To enable dengue sE protein expression, we included disulfide stabilized dimers with sequences derived from Cambodian patient isolates. To stabilize the dimer, 3 mutations were introduced to create 3 disulfide bonds, including one mutation in the fusion loop. Hence, this locked dimer does not expose the fusion loop (32). We included this notion in the discussion of the manuscript: “Comparing the binding profiles of antibodies secreted by single IgG-SC for sE-dimer or monomers allowed us to discern the epitope specificity; IgG-SC with specificity only to monomeric sE but not sE-dimer secrete most probably antibodies targeting the FL, whereas IgG-SC with specificity only to sE-dimer but not sE-monomer secrete most probably antibodies targeting quaternary epitopes, and IgG-SC with specificity to both monomeric sE and sE-dimers secrete EDIII- or EDII-specific antibodies.”

**Figure resubmission:**

While revising your submission, we strongly recommend that you use PLOS’s NAAS tool ([https://ngplosjournals.pagemajik.ai/artanalysis](https://apc01.safelinks.protection.outlook.com/?url=https%3A%2F%2Furldefense.com%2Fv3%2F__https%3A%2Fngplosjournals.pagemajik.ai%2Fartanalysis__%3B!!JFdNOqOXpB6UZW0!q5ejiA4Q3UYpPpqRDMomU0_Q3lWfh9p0k48TKsTOrmUpDkfJgj6wQdPO3tOJhq1O84AkMgj4i0KwWjPA-qI3xw%24&data=05%7C02%7Ctcantaert%40pasteur-kh.org%7C4bd884d9d21d4949a74b08deb4b41683%7C5a088b0e603f4d07a23c78b4a37af7f8%7C0%7C0%7C639146883763721188%7CUnknown%7CTWFpbGZsb3d8eyJFbXB0eU1hcGkiOnRydWUsIlYiOiIwLjAuMDAwMCIsIlAiOiJXaW4zMiIsIkFOIjoiTWFpbCIsIldUIjoyfQ%3D%3D%7C0%7C%7C%7C&sdata=t0VxuKkFDx%2BUeQMs8w6Jx9RhRDJfL1Q5wlMAWHaAq8g%3D&reserved=0)) to test your figure files. NAAS can convert your figure files to the TIFF file type and meet basic requirements (such as print size, resolution), or provide you with a report on issues that do not meet our requirements and that NAAS cannot fix.

After uploading your figures to PLOS’s NAAS tool - [https://ngplosjournals.pagemajik.ai/artanalysis](https://apc01.safelinks.protection.outlook.com/?url=https%3A%2F%2Furldefense.com%2Fv3%2F__https%3A%2Fngplosjournals.pagemajik.ai%2Fartanalysis__%3B!!JFdNOqOXpB6UZW0!q5ejiA4Q3UYpPpqRDMomU0_Q3lWfh9p0k48TKsTOrmUpDkfJgj6wQdPO3tOJhq1O84AkMgj4i0KwWjPA-qI3xw%24&data=05%7C02%7Ctcantaert%40pasteur-kh.org%7C4bd884d9d21d4949a74b08deb4b41683%7C5a088b0e603f4d07a23c78b4a37af7f8%7C0%7C0%7C639146883763738851%7CUnknown%7CTWFpbGZsb3d8eyJFbXB0eU1hcGkiOnRydWUsIlYiOiIwLjAuMDAwMCIsIlAiOiJXaW4zMiIsIkFOIjoiTWFpbCIsIldUIjoyfQ%3D%3D%7C0%7C%7C%7C&sdata=zmGju%2B%2B%2F4vJTYwPr%2FIHHyVvVHpReeGMq2Zo1ai0%2FZDQ%3D&reserved=0), NAAS will process the files provided and display the results in the "Uploaded Files" section of the page as the processing is complete. If the uploaded figures meet our requirements (or NAAS is able to fix the files to meet our requirements), the figure will be marked as "fixed" above. If NAAS is unable to fix the files, a red "failed" label will appear above. When NAAS has confirmed that the figure files meet our requirements, please download the file via the download option, and include these NAAS processed figure files when submitting your revised manuscript.
