# Supplementary material for: Quantitative and qualitative differences in anti-Envelope IgG-secreting cells distinguish subclinical and hospitalized dengue in a Cambodian pediatric cohort
Source: PLoS Pathog. 2026 Oct 1;22(10):e1014552. doi: 10.1371/journal.ppat.1014552 (PMC13649127; doi:10.1371/journal.ppat.1014552)
Supplement: Peer review history

# Peer review: Decision Letter 0

**DOI:** 10.1371/journal.ppat.1014552.r001

**Authors:** Sonja Best, Adam Waickman

17 May 2026

Quantitative and qualitative differences in anti-Evelope IgG-secreting cells distinguish subclinical and hospitalized dengue in a Cambodian pediatric cohort

PLOS Pathogens

Dear Dr. Cantaert,

Thank you for submitting your manuscript to PLOS Pathogens. After careful consideration, we feel that it has merit but does not fully meet PLOS Pathogens's publication criteria as it currently stands. Therefore, we invite you to submit a revised version of the manuscript that addresses the points raised during the review process.

Please submit your revised manuscript by Jul 16 2026 11:59PM. If you will need more time than this to complete your revisions, please reply to this message or contact the journal office at plospathogens@plos.org. When you're ready to submit your revision, log on to https://www.editorialmanager.com/ppathogens/ and select the 'Submissions Needing Revision' folder to locate your manuscript file.

Please include the following items when submitting your revised manuscript:

* A letter that responds to each point raised by the editor and reviewer(s). You should upload this letter as a separate file labeled 'Response to Reviewers'. This file does not need to include responses to any formatting updates and technical items listed in the 'Journal Requirements' section below.

* A marked-up copy of your manuscript that highlights changes made to the original version. You should upload this as a separate file labeled 'Revised Manuscript with Track Changes'.

* An unmarked version of your revised paper without tracked changes. You should upload this as a separate file labeled 'Manuscript'.

As the corresponding author, your ORCID iD is verified in the submission system and will appear in the published article. PLOS supports the use of ORCID, and we encourage all coauthors to register for an ORCID iD and use it as well. Please encourage your coauthors to verify their ORCID iD within the submission system before final acceptance, as unverified ORCID iDs will not appear in the published article. Only the individual author can complete the verification step; PLOS staff cannot verify ORCID iDs on behalf of authors.

We look forward to receiving your revised manuscript.

Kind regards,

Adam T. Waickman

Academic Editor

PLOS Pathogens

Sonja Best

Section Editor

PLOS Pathogens

Sumita Bhaduri-McIntosh

Editor-in-Chief

PLOS Pathogens

orcid.org/0000-0003-2946-9497

Michael Malim

PLOS Pathogens

orcid.org/0000-0002-7699-2064

**Journal Requirements:**

If the reviewer comments include a recommendation to cite specific previously published works, please review and evaluate these publications to determine whether they are relevant and should be cited. There is no requirement to cite these works unless the editor has indicated otherwise.

1) Please ensure that the CRediT author contributions listed for every co-author are completed accurately and in full.

At this stage, the following Authors/Authors require contributions: Amandine Trouchet, Lay Sokchea, Matteo Broketa, Pablo Canales-herreias, Angga Perima, Bruno IANNASCOLI, Sotheary Sann, Borita Heng, Ly Sowath, Veasna Duong, Laura Walker, Patrick England, Giovanna Barba-Spaeth, Tineke Cantaert, and Pierre Bruhns. Please ensure that the full contributions of each author are acknowledged in the "Add/Edit/Remove Authors" section of our submission form.

The list of CRediT author contributions may be found here: https://journals.plos.org/plospathogens/s/authorship#loc-author-contributions

2) Please provide an Author Summary. This should appear in your manuscript between the Abstract (if applicable) and the Introduction, and should be 150-200 words long. The aim should be to make your findings accessible to a wide audience that includes both scientists and non-scientists. Sample summaries can be found on our website under Submission Guidelines:

https://journals.plos.org/plospathogens/s/submission-guidelines#loc-parts-of-a-submission

3) Please upload all main figures as separate Figure files in .tif or .eps format. For more information about how to convert and format your figure files please see our guidelines:

https://journals.plos.org/plospathogens/s/figures

4) We have noticed that you have uploaded Supporting Information files, but you have not included a list of legends. Please add a full list of legends for your Supporting Information files after the references list.

5) We note that  1A, 2A, and S1A are  created through BioRender. Please confirm that you hold a Premium account and provide a pdf copy of the CC BY 4.0 Licence as provided by BioRender. For instructions on how to generate a CC BY 4.0 license for your figure, please see the guidelines here: https://help.biorender.com/hc/en-gb/articles/21282341238045-Publishing-in-open-access-resources

Please ensure the figure legend contains appropriate attribution text & a link to biorender.com

If you are using the free assets from BioRender, we are unable to publish these images as they are licenced under a stricter licence than CC BY 4.0. In this case we ask you to remove the BioRender images and replace them with open source alternatives.

See these open source resources you may use to replace images / clip-art:

- https://bioart.niaid.nih.gov/

- https://bioicons.com/

- https://healthicons.org/

- https://scidraw.io/

- https://reactome.org/icon-lib

- https://www.phylopic.org/images

- https://journals.plos.org/plosbiology/article?id=10.1371/journal.pbio.3002395

6) In the online submission form, you indicated that Any additional data (assay microscopy images and droplet detections with beadline intensity measurements) are available from the corresponding author upon request.. All PLOS journals now require all data underlying the findings described in their manuscript to be freely available to other researchers, either

1. In a public repository

2. Within the manuscript itself

3. Uploaded as supplementary information.

This policy applies to all data except where public deposition would breach compliance with the protocol approved by your research ethics board. If your data cannot be made publicly available for ethical or legal reasons (e.g., public availability would compromise patient privacy), please explain your reasons by return email and your exemption request will be escalated to the editor for approval. Your exemption request will be handled independently and will not hold up the peer review process, but will need to be resolved should your manuscript be accepted for publication. One of the Editorial team will then be in touch if there are any issues.

7) Please amend your detailed Financial Disclosure statement. This is published with the article. It must therefore be completed in full sentences and contain the exact wording you wish to be published.

1) State the initials, alongside each funding source, of each author to receive each grant. For example: "This work was supported by the National Institutes of Health (####### to AM; ###### to CJ) and the National Science Foundation (###### to AM)."

2) State what role the funders took in the study. If the funders had no role in your study, please state: "The funders had no role in study design, data collection and analysis, decision to publish, or preparation of the manuscript."

3) If any authors received a salary from any of your funders, please state which authors and which funders..

If you did not receive any funding for this study, please simply state: u201cThe authors received no specific funding for this work.u201d

**Reviewers' Comments:**

Reviewer's Responses to Questions

**Part I - Summary**

Please use this section to discuss strengths/weaknesses of study, novelty/significance, general execution and scholarship.

Reviewer #1: This an interesting study investigating how antibody responses differ between subclinical and hospitalized dengue infections, focusing on IgG-secreting cells (IgG-SC) at the single-cell level. Using a droplet-based microfluidic platform, the authors analyze over 3,300 individual cells from a pediatric cohort to measure antibody secretion, specificity, affinity, and cross-reactivity toward dengue envelope (E) protein antigens. They report that while hospitalized patients exhibit a higher proportion of dengue antibodies, these antibodies are of significantly lower affinity to the dimeric quaternary form of E protein compared to those from subclinical cases. Whereas hospitalized patients predominantly produce monomer-reactive antibodies, which were previously linked to poor neutralization and antibody enhancement of infection. The findings offer an important conceptual advance by linking disease severity not to the magnitude of the antibody response, but to its qualitative features; specifically, affinity and epitope targeting. The observation that subclinical cases harbor rare but potentially protective quaternary epitope–specific antibodies provide support for vaccine strategies that aim to elicit such responses. The study also reinforces the idea that high-frequency, low-affinity, and monomer-directed responses may contribute to disease pathology.

Reviewer #2: Bruhns and coworkers used the very interesting and powerful DropMap technology they had developed earlier for the single cell level analysis of antibody secretion and affinity to characterize the response to subclinical or hospitalized post‑primary dengue (DENV1 or DENV2) in a pediatric cohort. The key finding is that hospitalized patients show a higher fraction of DENV‑specific ASCs which however produce lower‑affinity antibodies, are skewed toward monomer (sE) binding but not towards protective quaternary epitopes. The work is technically innovative, conceptually interesting and the data intriguing.

**********

**Part II – Major Issues: Key Experiments Required for Acceptance**

Please use this section to detail the key new experiments or modifications of existing experiments that should be absolutely required to validate study conclusions.

Generally, there should be no more than 3 such required experiments or major modifications for a "Major Revision" recommendation. If more than 3 experiments are necessary to validate the study conclusions, then you are encouraged to recommend "Reject".

Reviewer #1: 1. A substantial number of cells were analyzed in this study, with results presented as pooled data across all cells and all donors in the same group. However, given the relatively modest cohort size (6 and 9 donors), pooling across individuals may introduce bias. This is particularly relevant if certain donors contribute disproportionately larger numbers of cells, potentially skewing the overall trends. The authors acknowledge this limitation in the Discussion, noting that fewer cells were obtained from hospitalized samples compared to subclinical. Nonetheless, to ensure that the observed reported patterns and differences between groups are not driven by only a small subset of individuals it is important to provide a clear breakdown of the number of cells analyzed per donor. If substantial cell number differences exist between individuals, this needs to be addressed and if this is the case, normalization or down-sampling approaches should be considered to reduce overrepresentation from donors contributing more cells. More broadly, key comparisons including IgG secretion, antigen binding, frequency of positive cells, affinity, and epitope specificity, should be also analyzed at the individual donor level to strengthen the confidence of the main findings and ensure that the findings are representative of the cohort as a whole and not driven by only a few donors withing each group.

Reviewer #2: A few points need to be addressed before publication:

1. The distinction between “subclinical” and “hospitalized” dengue is central to the paper but not well defined. How many subclinical cases were completely asymptomatic vs mildly symptomatic? Did any meet WHO “dengue” criteria but did not require hospitalization?

2. Cell numbers in hospitalized patients are lower. To what extent this factor could have influenced the key conclusions here? Also the effect of freeze thaw on plasma blast viability (which may be affected) by disease status should be at least discussed.

3. The findings regarding higher frequencies of ASCs producing sE specific antibodies as opposed to quaternary epitopes in hospitalized patients is extremely interesting and probably the most significant finding from this work. But then neither neutralization nor binding titers were different between the two groups. Perhaps a per patient correlation analysis of FRNT/MIA and DropMap parameters could be illuminating. Separately could it be that FRNT/MIA are strongly influenced by pre-existing circulating antibodies from LLPCs which would not be detected here? In any event at the very least this issue needs to be addressed in the Discussion.

4. The use three competing E‑derived antigens per droplet is a very important advance for DropMap. Please explain more clearly how completion influences the measured slope and KD values especially for cross reactive EDIII antibodies.

**********

**Part III – Minor Issues: Editorial and Data Presentation Modifications**

Please use this section for editorial suggestions as well as relatively minor modifications of existing data that would enhance clarity.

Reviewer #1: Minor comments:

2. Line 120:” Binding to anti-EDIII from DENV1 tended to be higher than the other serotypes, irrespective of the current infecting serotype, except for one individual with a strong EDIII DENV-4 binding preference (Figure 1B)”- this trend is not readily apparent in the current presentation of the figure. For example, samples S1, H2, and H3 appear to show comparable reactivity across all DENV antigens tested, making it difficult to discern the stated pattern. More broadly, panels B and C are challenging to interpret in their current format. It may be helpful to reorganize these panels to improve clarity (presenting the data as a heatmap could make relative differences and trends more immediately apparent). As it stands, extracting clear conclusions from these panels is not straightforward. Importantly, the authors need to include the appropriate negative control antigens to distinguish polyreactive serum responses from those that are specific to DENV or ZIKV.

3. In Figure 2, it is not clear whether the sE/sE-dimer positive IgG SCs shown in dark green indicate cells that bind both forms, only one form (and which one) or either one of the forms? Again, data representation can be improved.

4. The observation that approximately 10% of IgG-secreting cells from healthy donors show binding to DENV1/2 is somewhat unexpected and would benefit from further clarification. Could the authors comment on the nature of these responses? For example, do these cells represent low-affinity or polyreactive antibodies, or might this reflect background signal inherent to the assay? Related to this, it would be helpful to better understand how binding was defined. If this signal represents baseline assay noise, should a more stringent threshold be applied to classify an IgG-secreting cell as “binding”? Providing additional detail on how the signal was measured, including the criteria and thresholds used to define positivity, would greatly improve interpretability. Overall, further clarification of these points would help distinguish true antigen-specific responses from background reactivity.

5. It seems that the IgG secretion rate in total IgG is not different from the secretion rate in DENV binding IgG. Is it possible that these differences are patient specific/intrinsic and have no relation to the infection severity?

6. Line 177: “The percentage of IgG-SC binding to at least one DENV sE protein among all IgG-SC was significantly lower in subclinical cases compared to hospitalized patients infected with DENV1 (41% vs 77%, respectively; 1.8-fold difference) and DENV2 (49 vs 61%, respectively; 1.2-fold difference) (Figure 2C)”. Can the authors explain what is the meaning of this? In general, it would be helpful to add a short conclusion sentence at the end of each Results section to increase clarity of the text.

7. The error bar in Figure 4S looks off set.

8. Why affinities in the hospitalized cases were not analyzed?

9. Figure 4B: representation could be improved to increase clarity. Number of subjects and number of cells need to be indicated. The method for statistical analysis needs to be indicated.

Reviewer #2: Minor: The authors used disulfide stabilized dimers however these can limit access to epitopes such as fusion loop compared to non-covalent dimers. This possible limitation should be mentioned in the Discussion.

**********

PLOS authors have the option to publish the peer review history of their article (what does this mean?). If published, this will include your full peer review and any attached files.

If you choose “no”, your identity will remain anonymous but your review may still be made public.

**Do you want your identity to be public for this peer review?**  For information about this choice, including consent withdrawal, please see our Privacy Policy.

Reviewer #1: No

Reviewer #2: No

**Figure resubmission:**

**Reproducibility:**

-->?>

# Author comment: Author response to Decision Letter 1

**DOI:** 10.1371/journal.ppat.1014552.r002

29 Jul 2026

Attachment Submitted filename: 20260707 Reviewers replies.docx

# Peer review: Decision Letter 1

**DOI:** 10.1371/journal.ppat.1014552.r003

**Authors:** Sonja Best, Adam Waickman

18 Aug 2026

Dear Dr Cantaert,

We are pleased to inform that your manuscript, "Quantitative and qualitative differences in anti-Envelope IgG-secreting cells distinguish subclinical and hospitalized dengue in a Cambodian pediatric cohort", has been editorially accepted for publication at PLOS Pathogens.

Before your manuscript can be formally accepted and sent to production, you will need to complete our formatting changes, which you will receive by email within a week. Please note that your manuscript will not be scheduled for publication until you have made the required changes.

IMPORTANT NOTES

(1) Copyediting and Proofreading: The corresponding author will receive a typeset proof for review, to ensure errors have not been introduced during production. Please review the PDF proof of your manuscript carefully, as this is the last chance to correct any errors. Please note that major changes, or those which affect the scientific understanding of the work, will likely cause delays to the publication date of your manuscript.

(2) Appropriate Figure Files: Please remove all name and figure # text from your figure files. Please also take this time to check that your figures are of high resolution, which will improve the readbility of your figures and help expedite your manuscript's publication. Please note that figures must have been originally created at 300dpi or higher. Do not manually increase the resolution of your files. For instructions on how to properly obtain high quality images, please review our Figure Guidelines, with examples at: http://journals.plos.org/plospathogens/s/figures.

We encourage you to use PACE (http://pace.apexcovantage.com/), a figure-checking tool which helps authors ensure all figures meet PLOS requirements so that figures can publish in the highest quality. PACE is a digital diagnostic and conversion tool for figure files. It will provide information about any failed check(s) and, if able, will automatically convert the figure into an acceptable file that passes quality checks.

To use PACE, you must first register as a user. Then, login and navigate to the UPLOAD tab, where you will find detailed instructions on how to use the tool.

(3) Striking Image: Please upload a striking still image to accompany your article if one is available (you can include a new image or an existing one from within your manuscript). Should your paper be accepted, this image will be considered for our monthly issue image and may also appear on our website to feature your article. Please upload this as a separate file, selecting "striking image" as the file type upon upload. Please also include a separate "Other" file with a caption, including credits and any potential copyright information. Please do not include the caption in the main article file. If your image is from someone other than yourself, please ensure that the artist has read and agreed to the terms and conditions of the Creative Commons Attribution License at http://journals.plos.org/plospathogens/s/content-license. Please note that PLOS cannot publish copyrighted images.

(4) Press Release or Related Media: If you or your institution will be preparing press materials for this manuscript, please inform our press team in advance at plospathogens@plos.org as soon as possible. We ask that you contact us within one week to plan ahead of our fast Production schedule. If you need to know your paper's publication date for related media purposes, you must coordinate with our press team, and your manuscript will remain under a strict press embargo until the publication date and time.

(5) Update your Profile Information: Now that your manuscript has been provisionally accepted, please log into Editorial Manager and update your profile, if needed. Go to http://www.editorialmanager.com/ppathogens, log in, and click on the "Update My Information" link at the top of the page. Please update your user information to ensure an efficient production and billing process.

(6) LaTeX users only: Our staff will ask you to upload a TEX file in addition to the PDF before the paper can be sent to typesetting, so please carefully review our Latex Guidelines http://journals.plos.org/plospathogens/s/latex in the meantime.

Thank you again for supporting Open Access publishing; we are looking forward to publishing your work in PLOS Pathogens.

Best regards,

Adam T. Waickman

Academic Editor

PLOS Pathogens

Sonja Best

Section Editor

PLOS Pathogens

Dominique Soldati-Favre

Editor-in-Chief

PLOS Pathogens

***********************************************************

Reviewer's Responses to Questions

**Part I - Summary**

Please use this section to discuss strengths/weaknesses of study, novelty/significance, general execution and scholarship.<br/><br/>

Reviewer #1: The authors have done an excellent job carrying out a thorough revision of the manuscript. They have addressed all my comments, by bringing in a statistician and carrying out additional, while also revising their conclusions to better reflect the scope and limitations of the data. This revised manuscript is substantially strengthened, and presents a more balanced and rigorous interpretation of the findings.

Reviewer #2: (No Response)

--------------------

**Part II – Major Issues: Key Experiments Required for Acceptance**

Please use this section to detail the key new experiments or modifications of existing experiments that should be absolutely required to validate study conclusions.<br/><br/>Generally, there should be no more than 3 such required experiments or major modifications for a "Major Revision" recommendation. If more than 3 experiments are necessary to validate the study conclusions, then you are encouraged to recommend "Reject".<br/><br/>

Reviewer #1: none

Reviewer #2: I am Satisfied with the author's response and edits to the manuscript. All issues have been addressed and the paper is no suitable for publication

--------------------

**Part III – Minor Issues: Editorial and Data Presentation Modifications**

Please use this section for editorial suggestions as well as relatively minor modifications of existing data that would enhance clarity.<br/><br/>

Reviewer #1: Two small comments:

1.The last sentence of the Abstract is not very clear and I suggest modifying or simplifying it. “These findings suggest that dengue severity may be linked to the inefficient generation of sufficient numbers of high-affinity, broadly reactive antigen-specific IgG-SC during post-primary infection.”

2.Line 216: “In conclusion, hospitalized patients displayed significantly lower numbers of IgG-SC, … ” I could not find any statistical analysis data supporting this conclusion. Figure 2C shows individual frequencies of IgG-SC per donor but no statistical significance is displayed there. For the left graph it does look like the subclinical donors have more IgG SC, but this is not the case in the right graph. In any case, stating “significantly lower numbers of” seems not the right phrasing.

Reviewer #2: No issues

--------------------

PLOS authors have the option to publish the peer review history of their article (what does this mean?). If published, this will include your full peer review and any attached files.

If you choose “no”, your identity will remain anonymous but your review may still be made public.

**Do you want your identity to be public for this peer review?**  For information about this choice, including consent withdrawal, please see our Privacy Policy.

Reviewer #1: No

Reviewer #2: No

# Editor report: Acceptance letter

**DOI:** 10.1371/journal.ppat.1014552.r004

**Authors:** Sonja Best, Adam Waickman

Dear Dr Cantaert,

We are delighted to inform you that your manuscript, "Quantitative and qualitative differences in anti-Envelope IgG-secreting cells distinguish subclinical and hospitalized dengue in a Cambodian pediatric cohort," has been formally accepted for publication in PLOS Pathogens.

We have now passed your article onto the PLOS Production Department who will complete the rest of the pre-publication process. All authors will receive a confirmation email upon publication.

Thank you again for supporting open-access publishing; we are looking forward to publishing your work in PLOS Pathogens.

Best regards,

Dominique Soldati-Favre

Editor-in-Chief

PLOS Pathogens
